# Supplementary figures and images for: Gut microbiota-derived gamma-aminobutyric acid from metformin treatment reduces hepatic ischemia/reperfusion injury through inhibiting ferroptosis
Source: eLife. 2024 Mar 15;12:RP89045. doi: 10.7554/eLife.89045 (PMC10942780; doi:10.7554/eLife.89045)

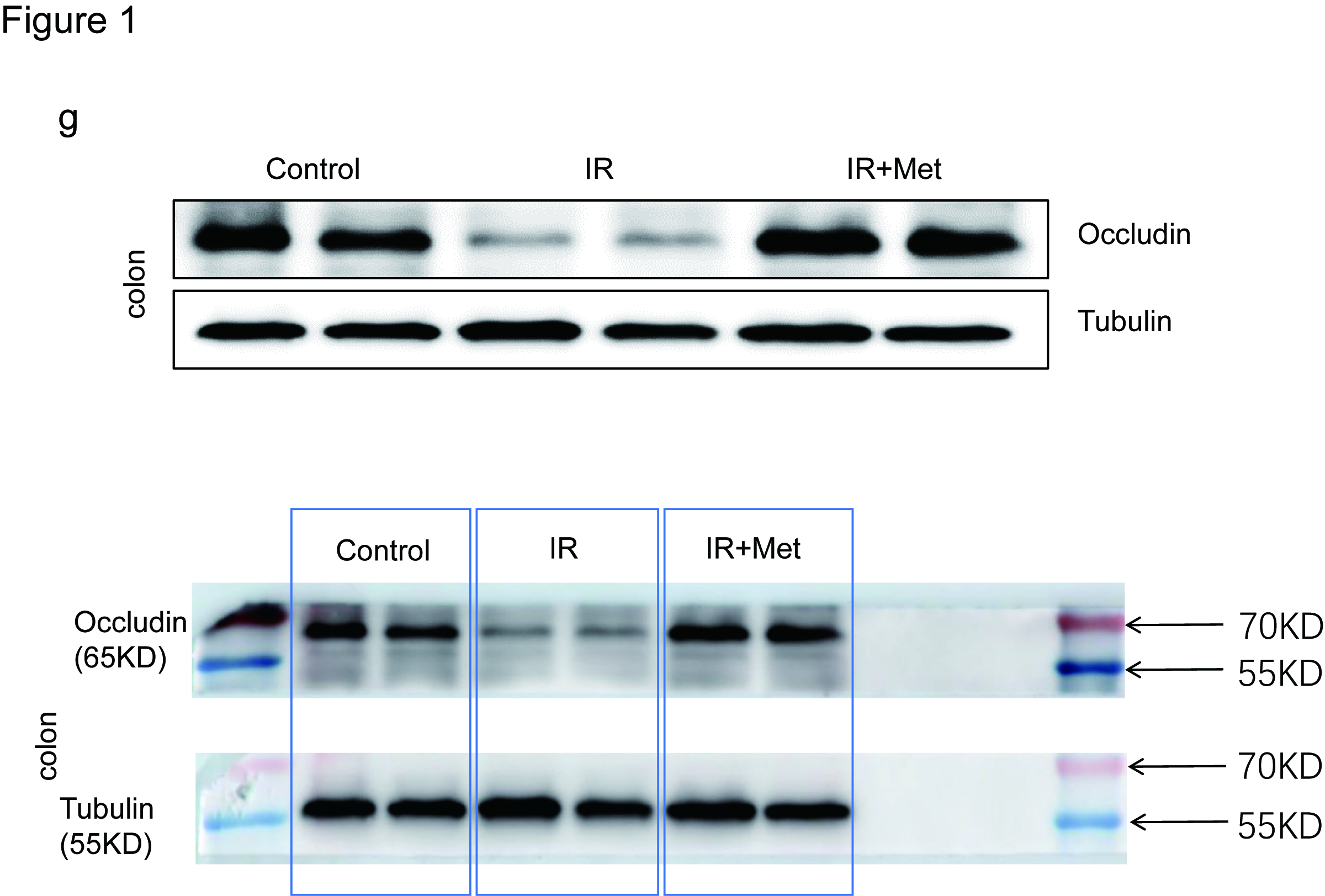

Supplement: Figure 1—source data 1. [file elife-89045-fig1-data1.zip › Figure 1-source data 1.tif]

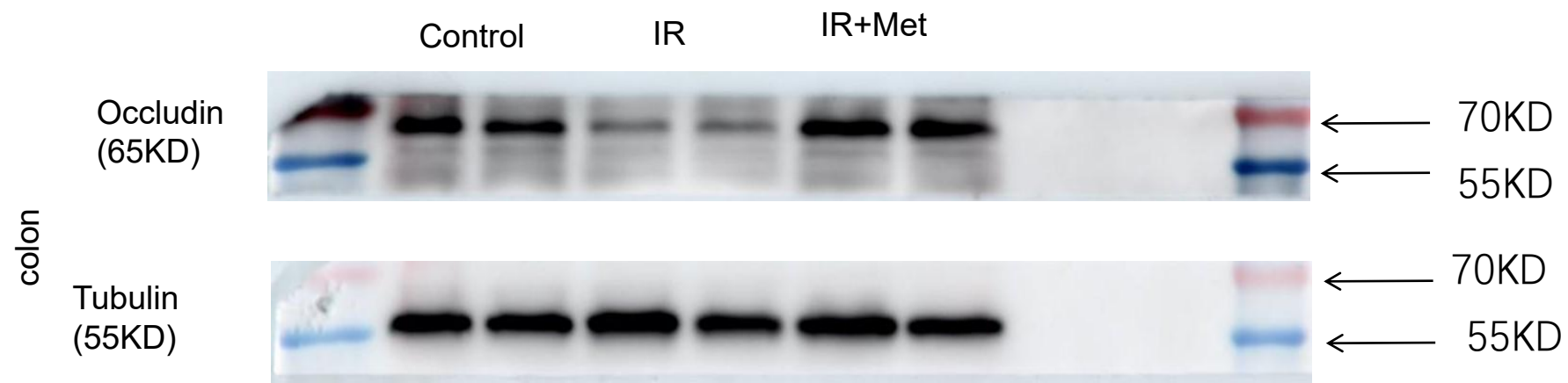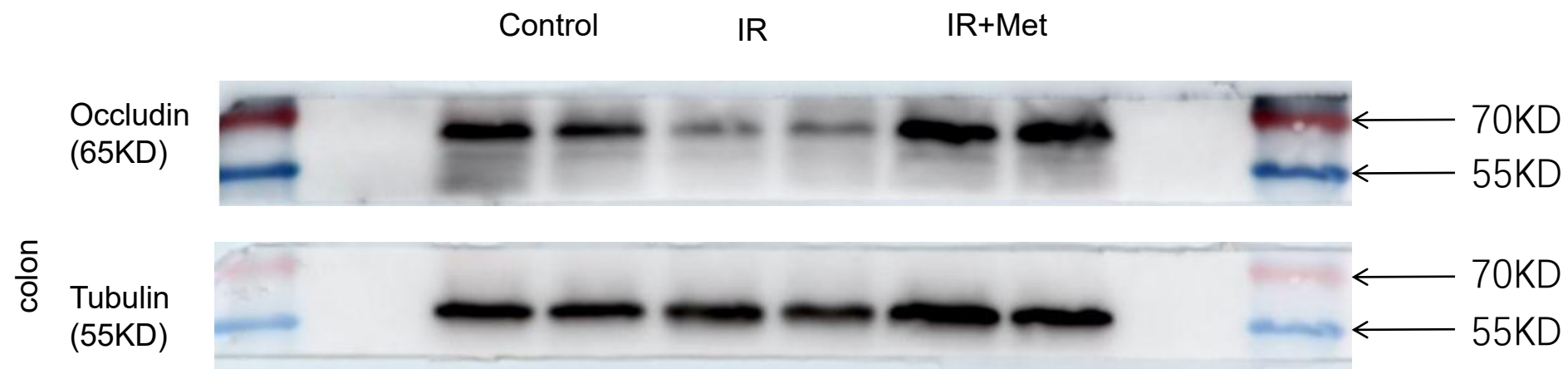

Supplement: Figure 1—source data 2. [file elife-89045-fig1-data2.zip › Figure 1-source data 2.pdf]

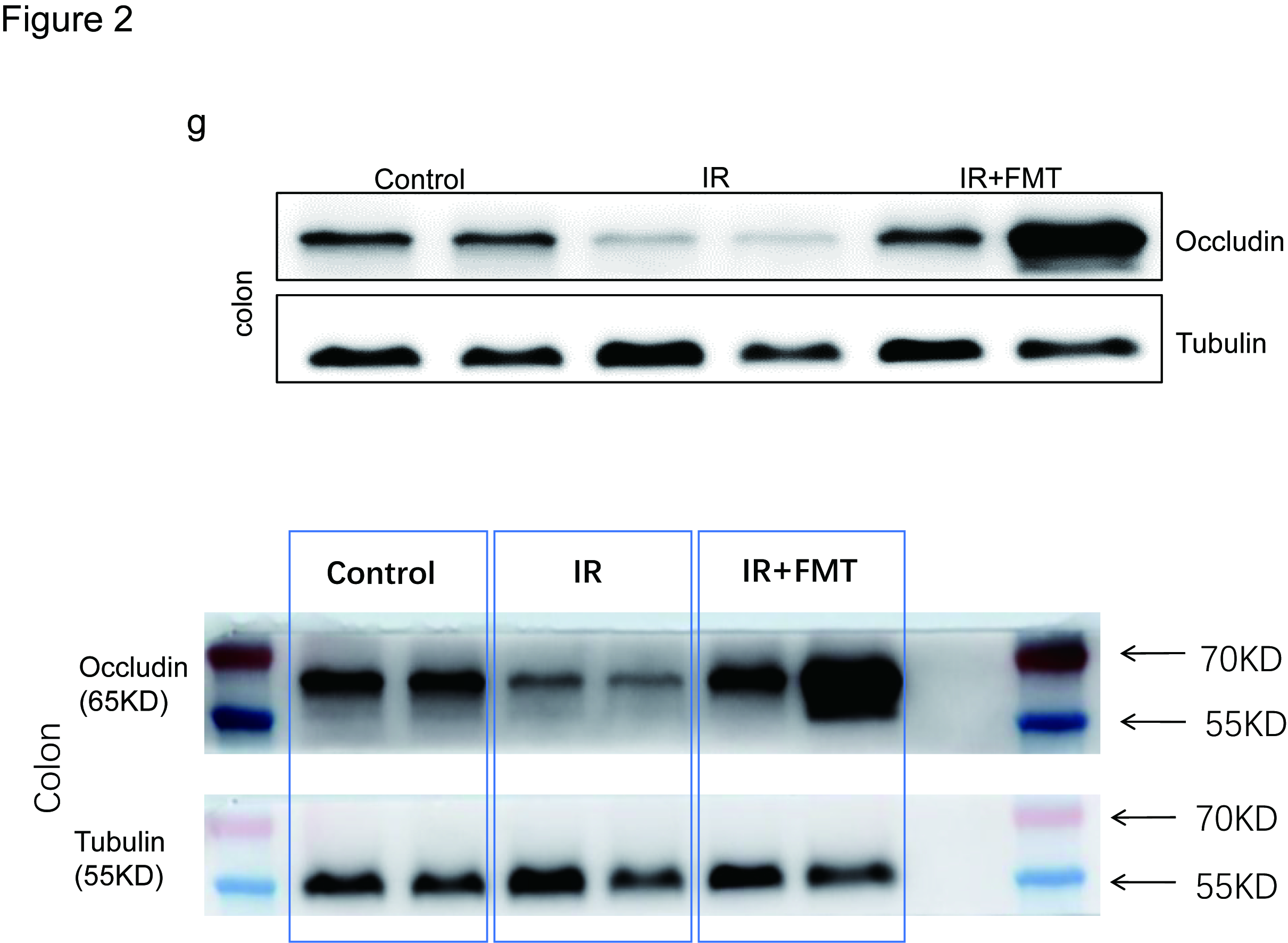

Supplement: Figure 2—source data 1. [file elife-89045-fig2-data1.zip › Figure 2-source data 1.tif]

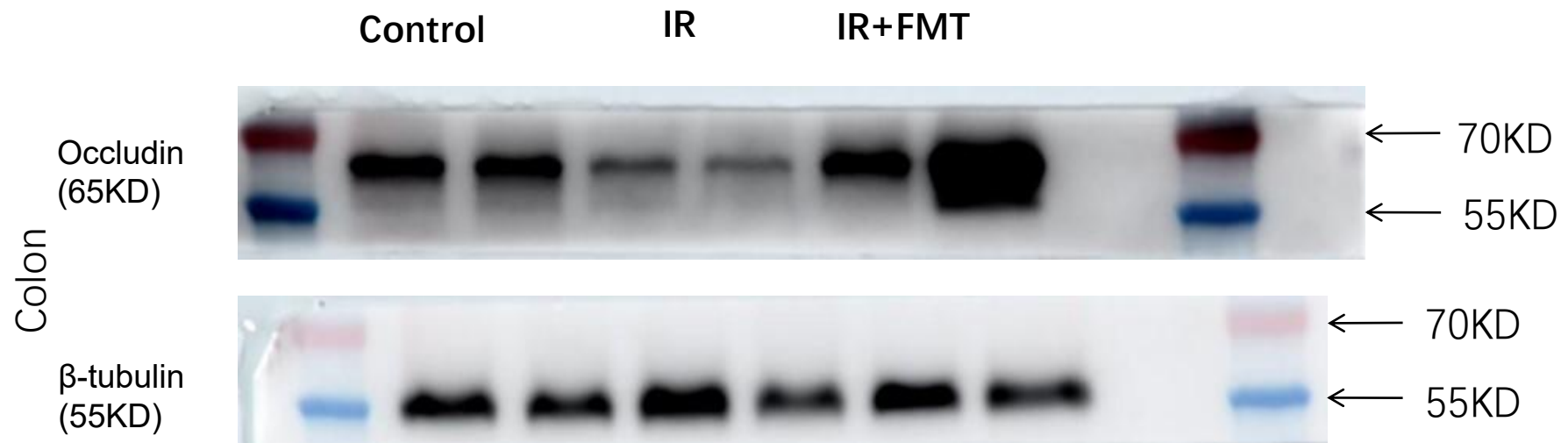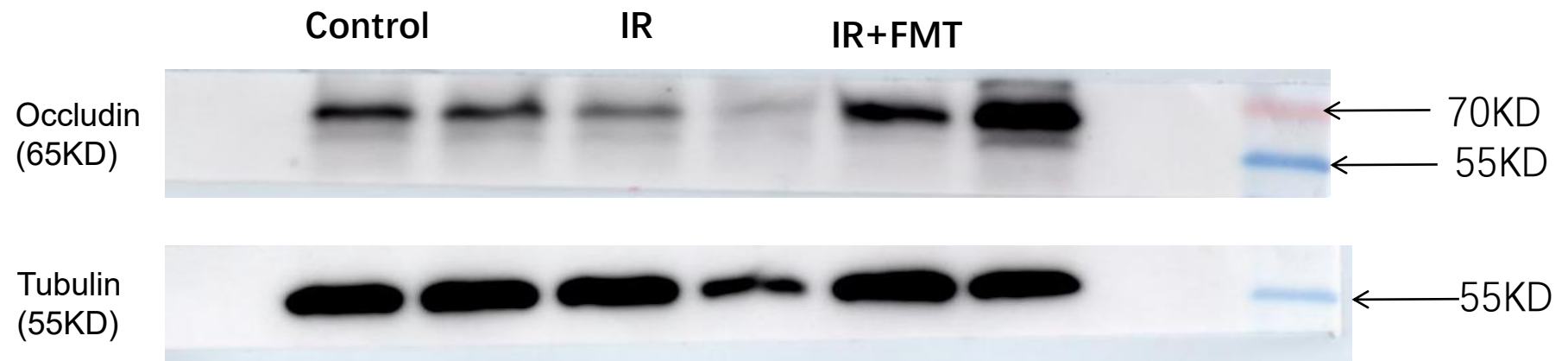

Supplement: Figure 2—source data 2. [file elife-89045-fig2-data2.zip › Figure 2-source data 2.pdf]

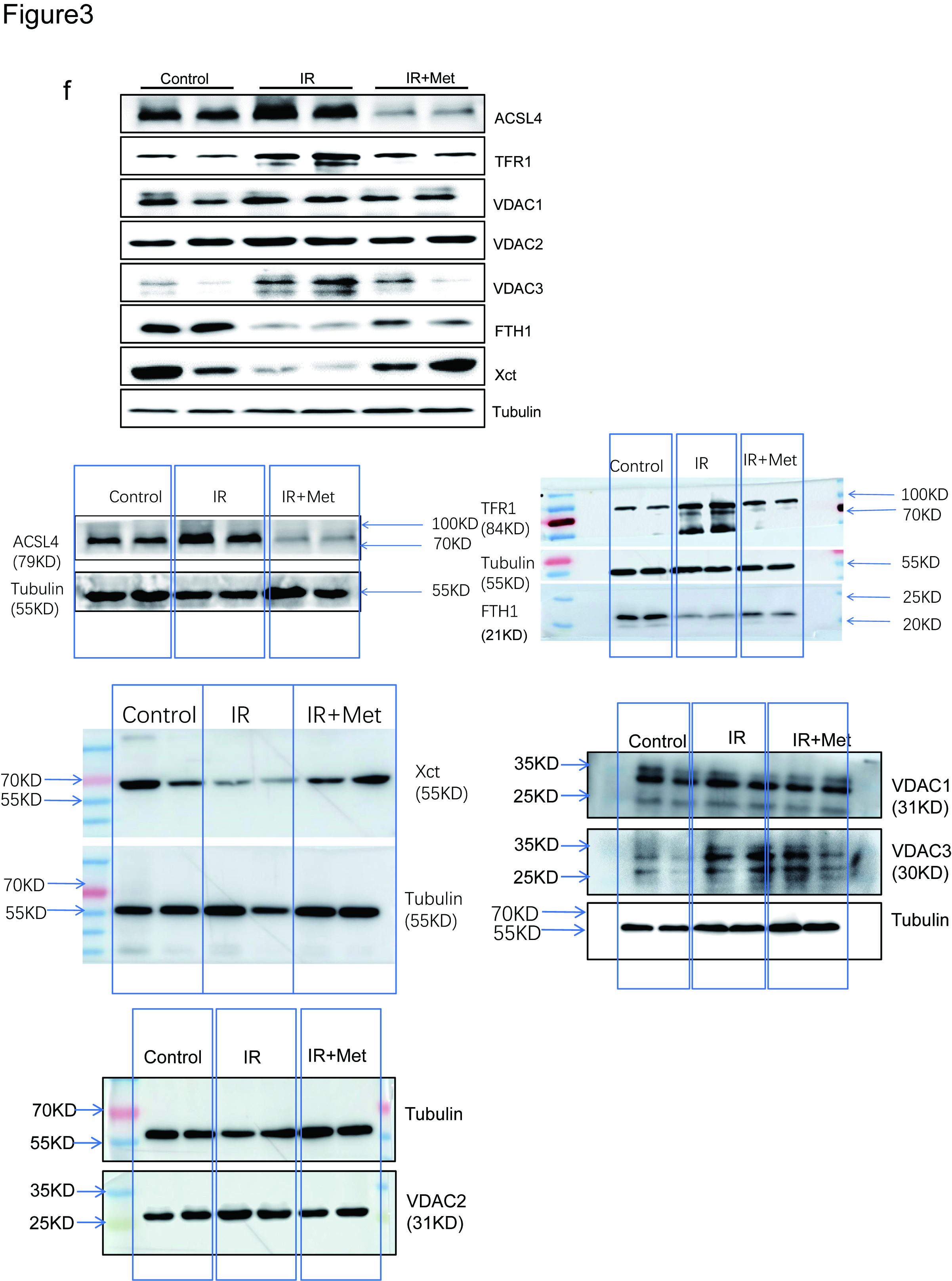

Supplement: Figure 3—source data 1. [file elife-89045-fig3-data1.zip › Figure 3-source data 1/Figure 3-source data 1.tif]

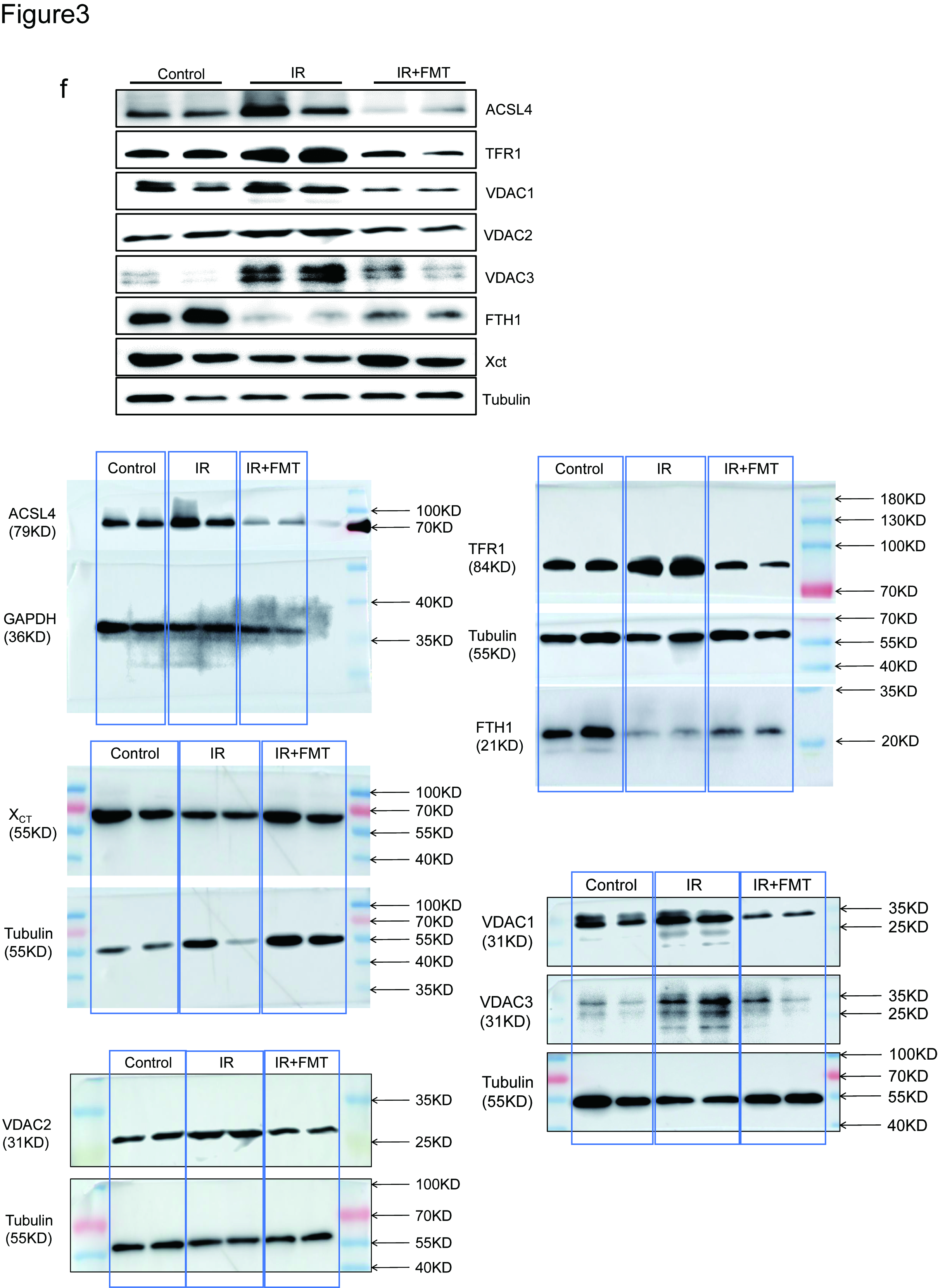

Supplement: Figure 3—source data 1. [file elife-89045-fig3-data1.zip › Figure 3-source data 1/Figure 3-source data 1-2.tif]

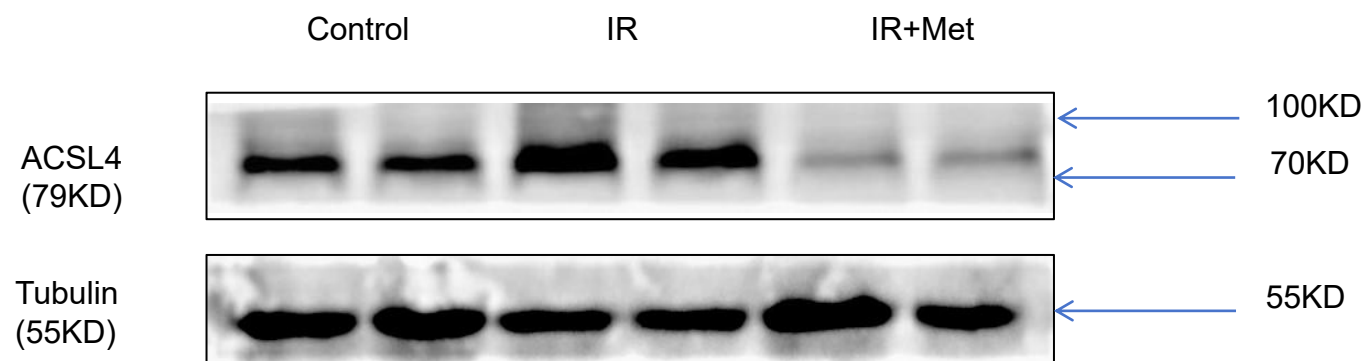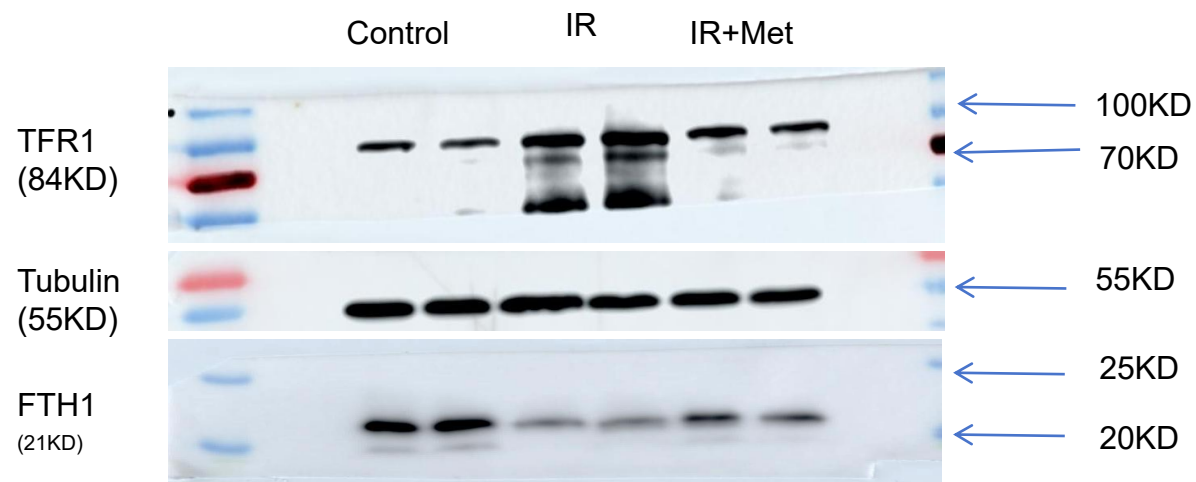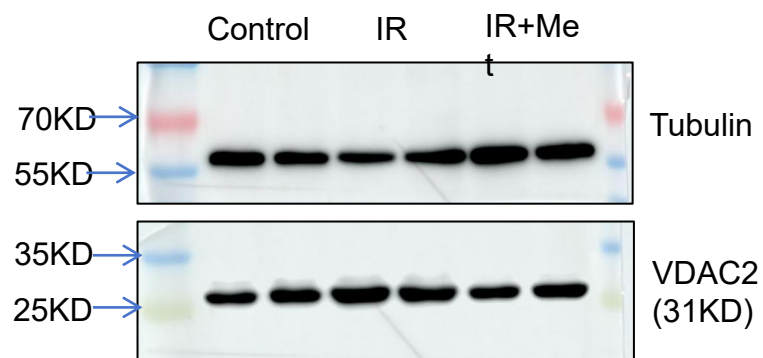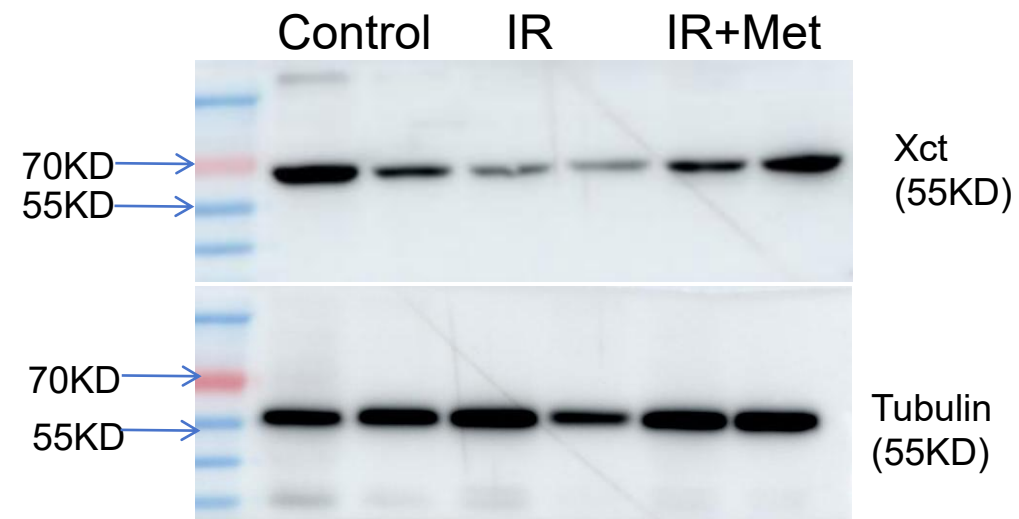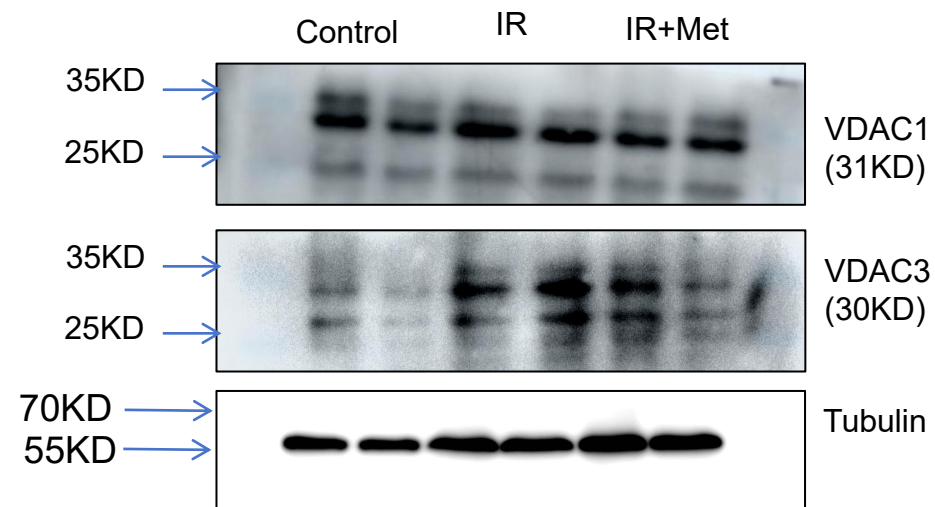

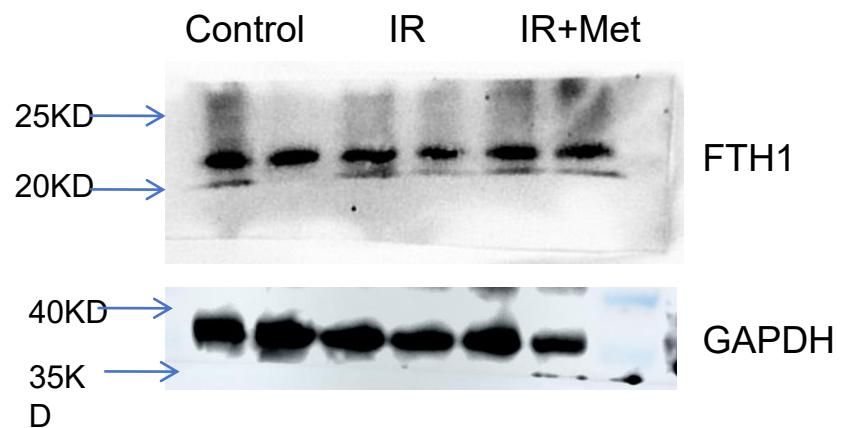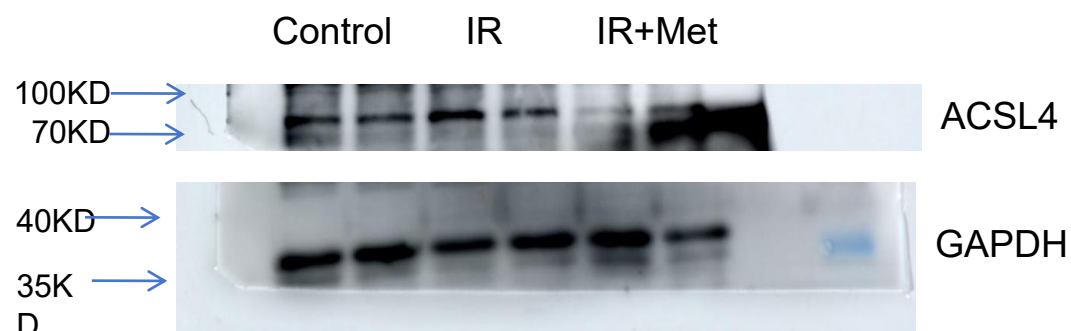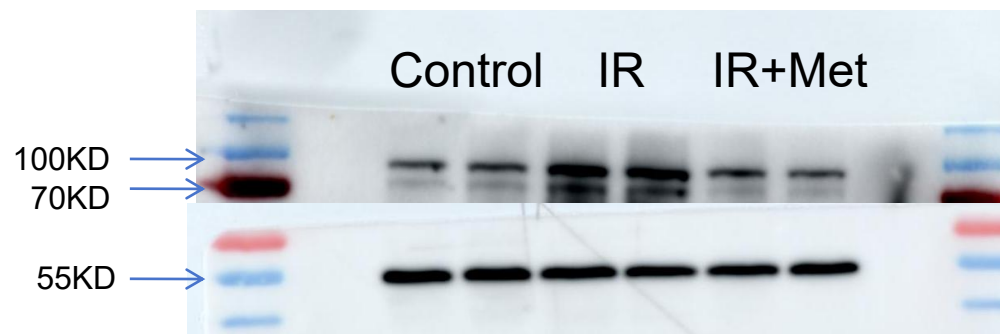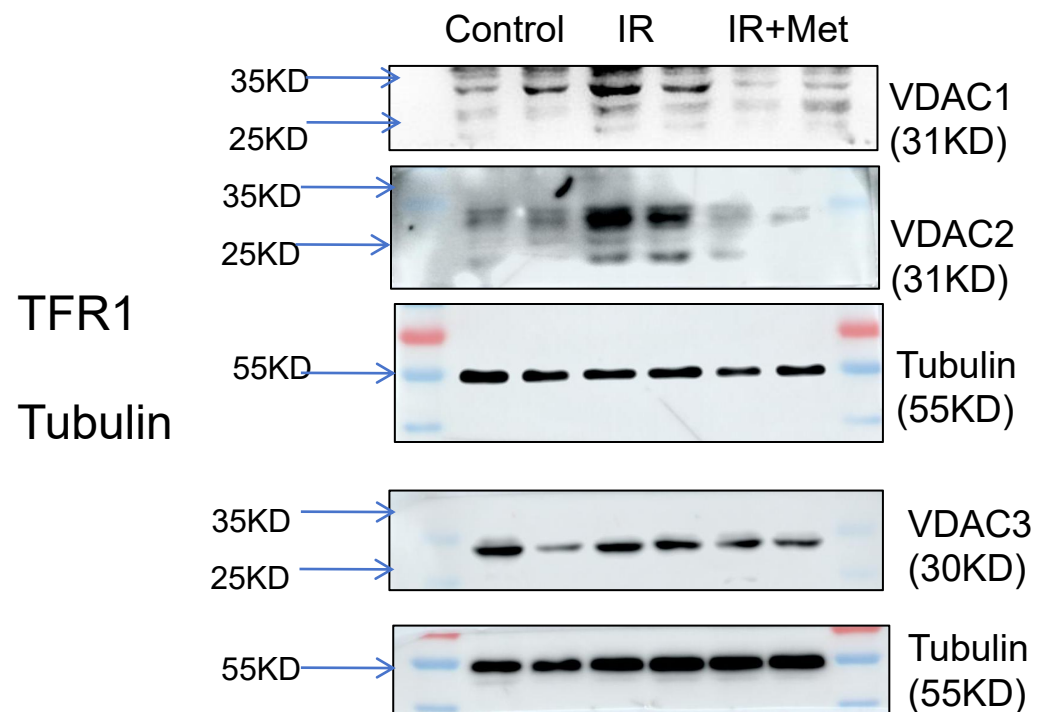

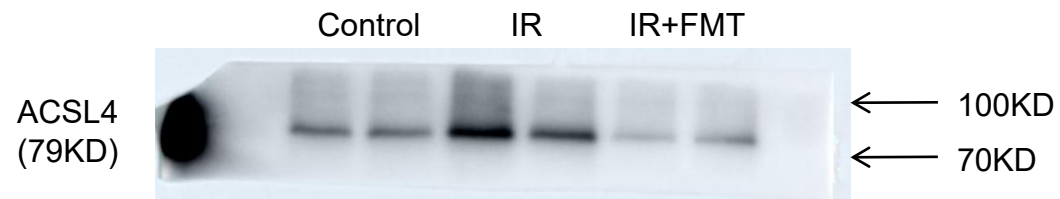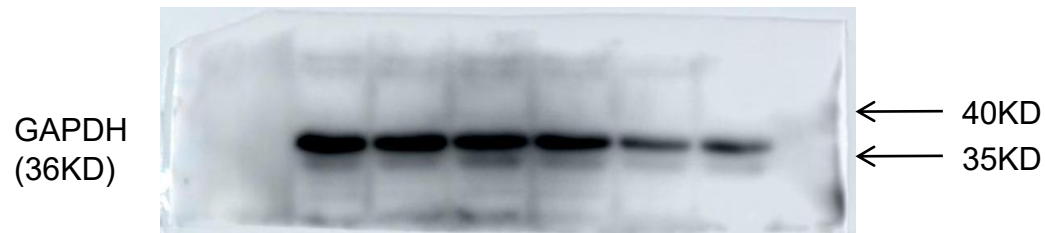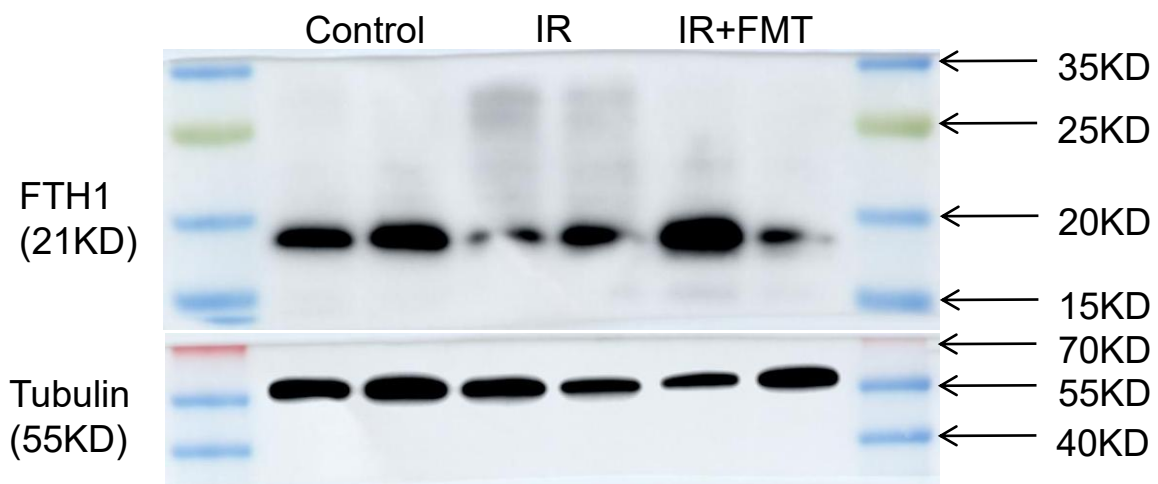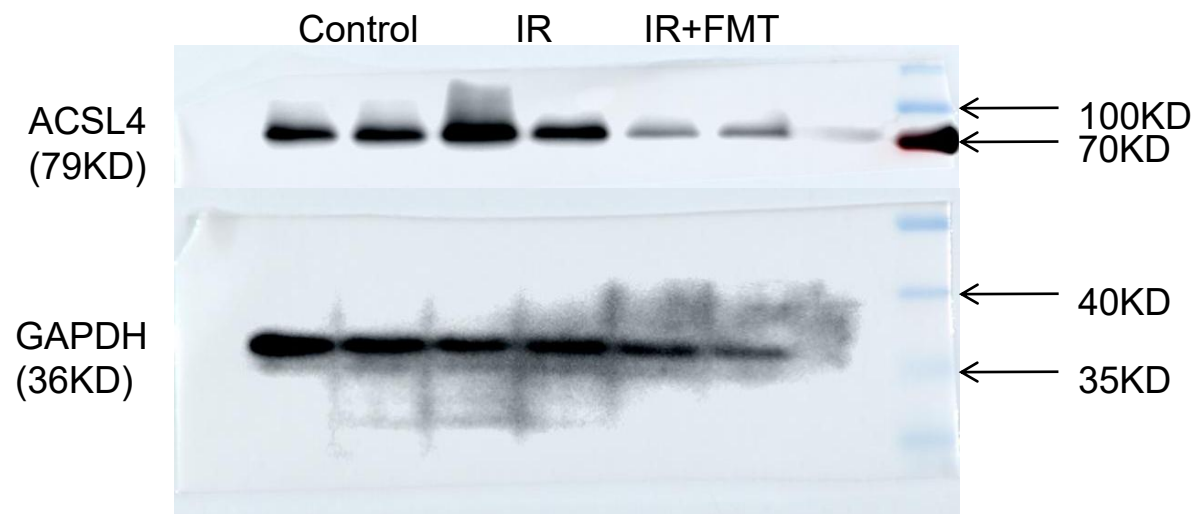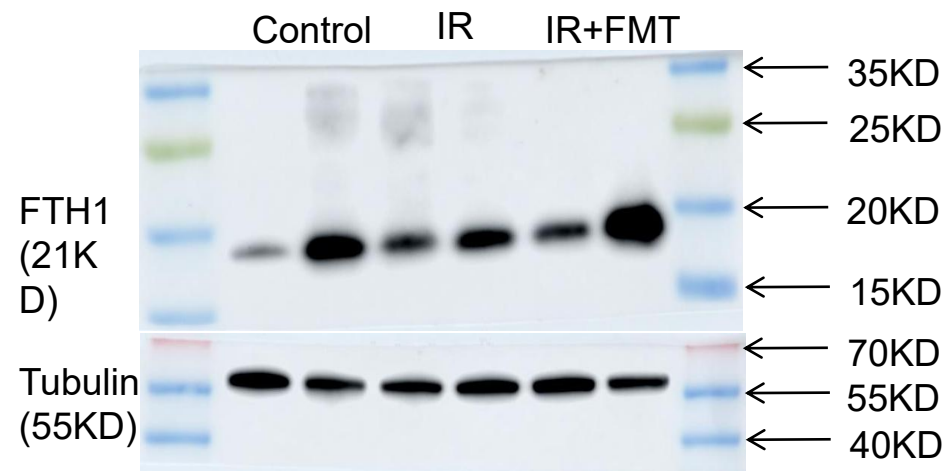

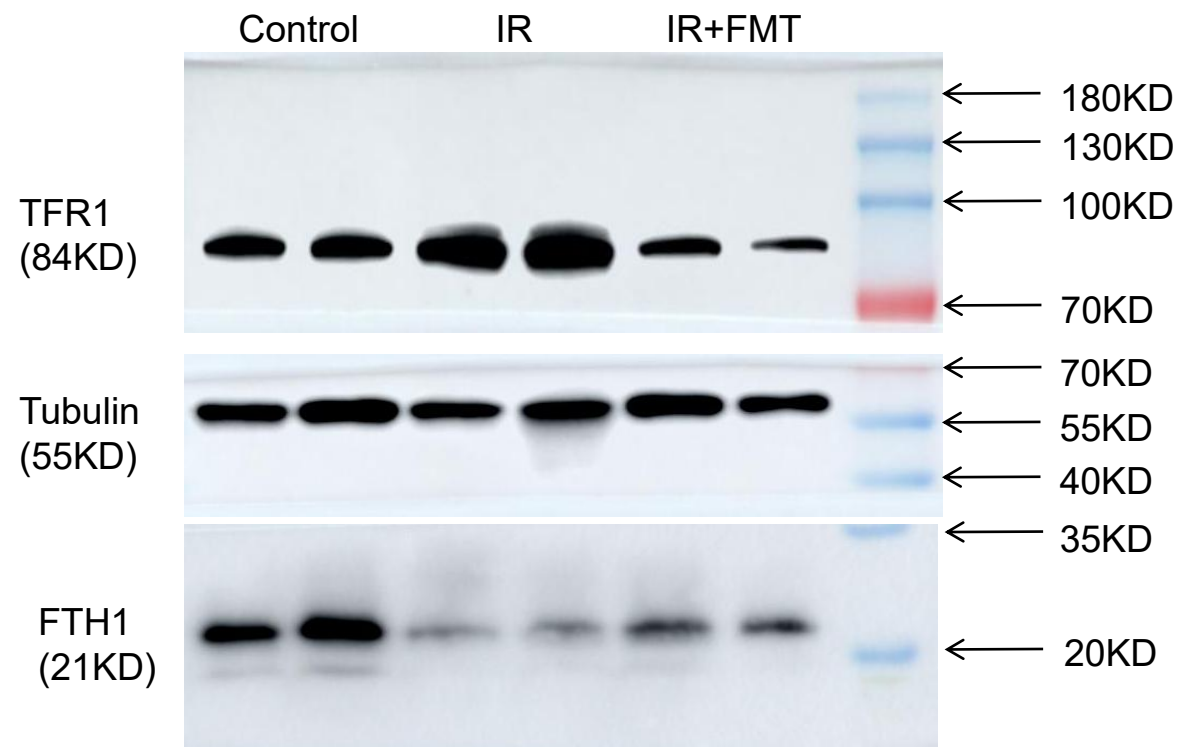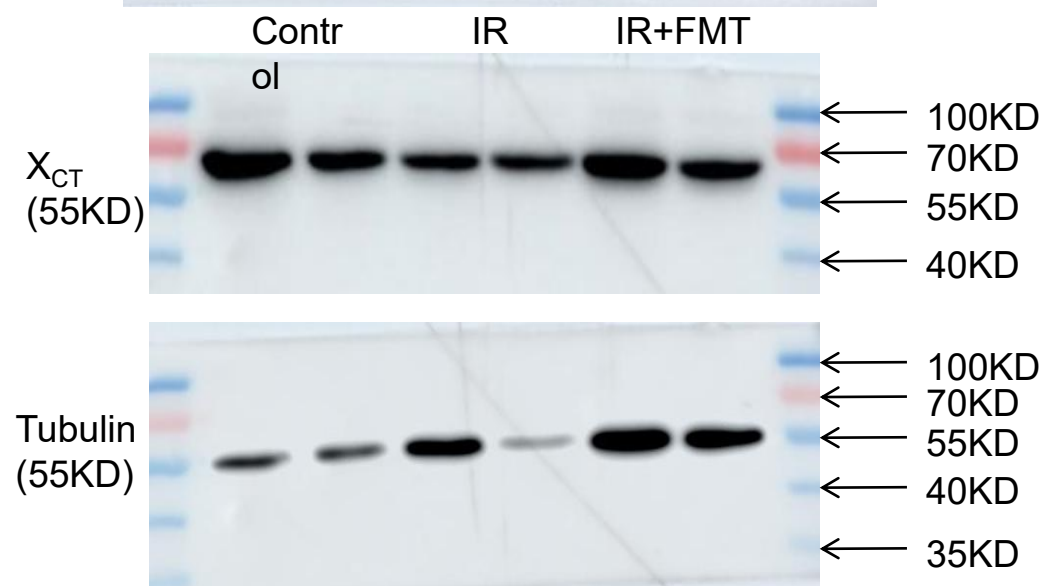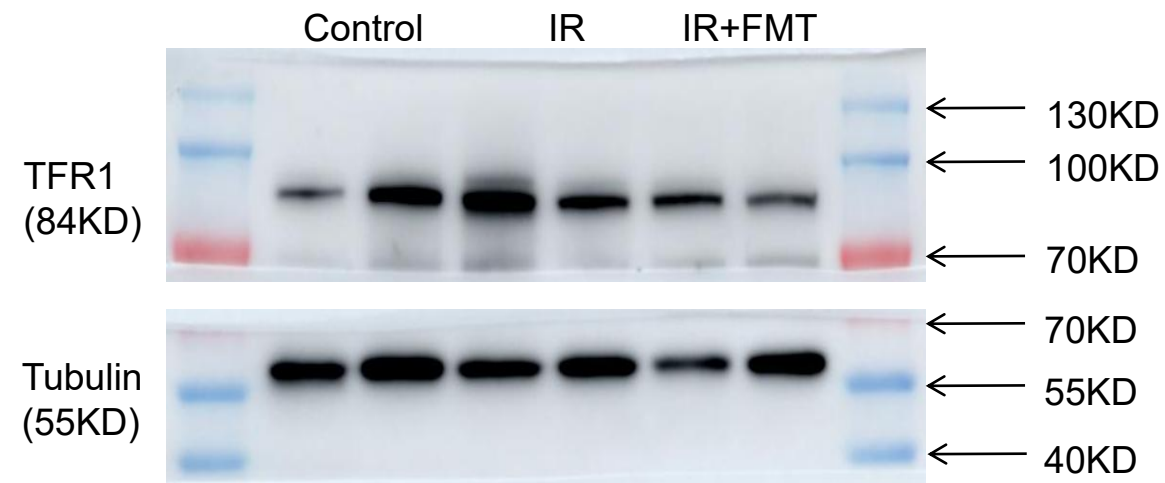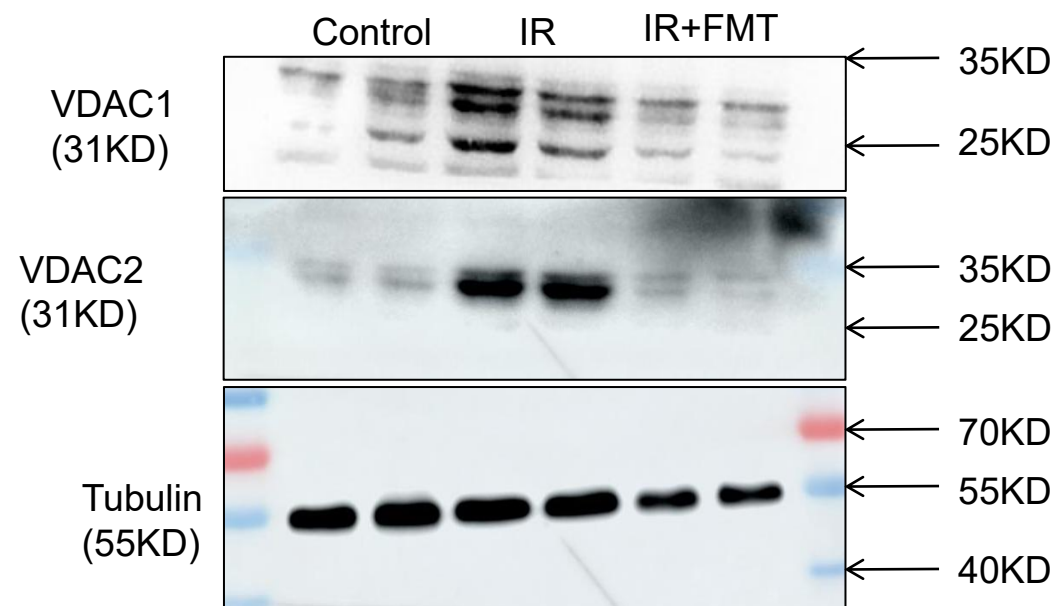

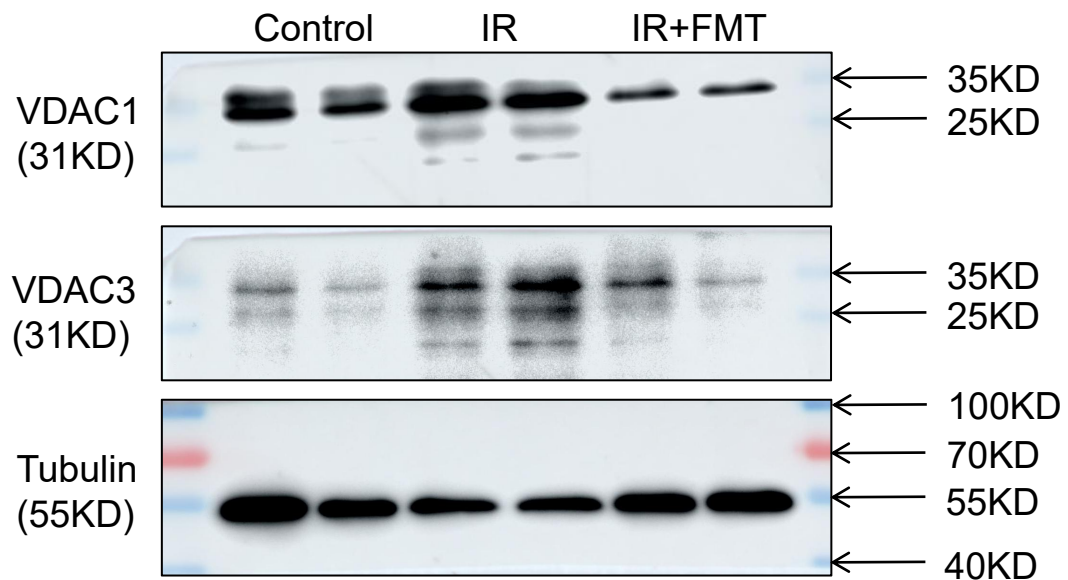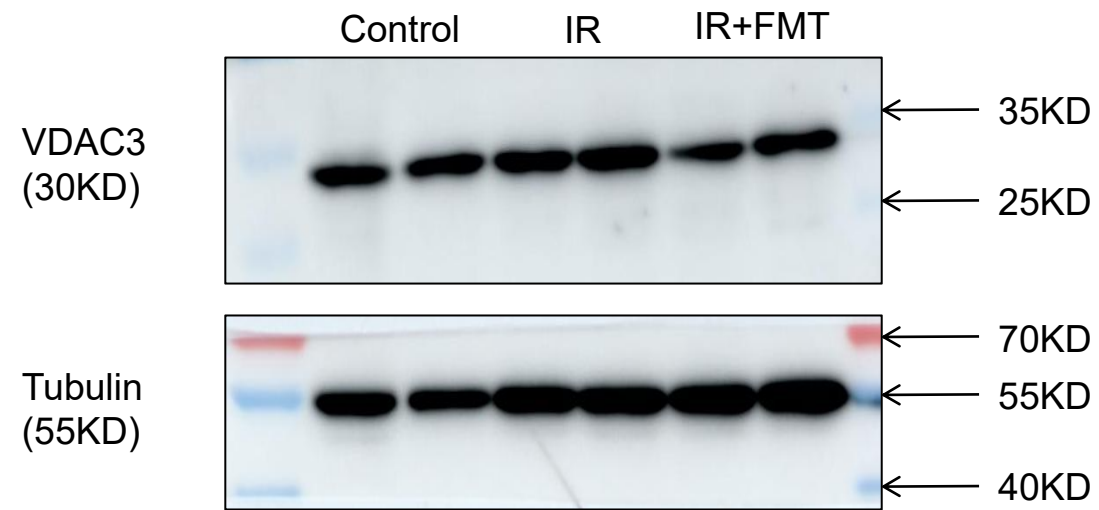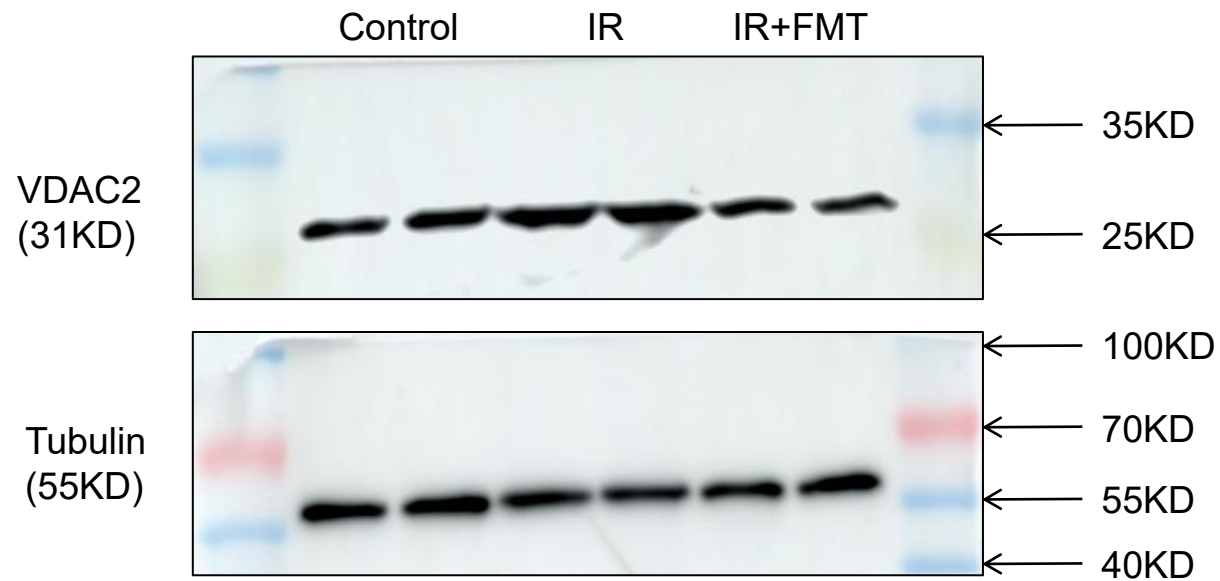

Supplement: Figure 3—source data 2. [file elife-89045-fig3-data2.zip › Figure 3-source data 2.pdf]

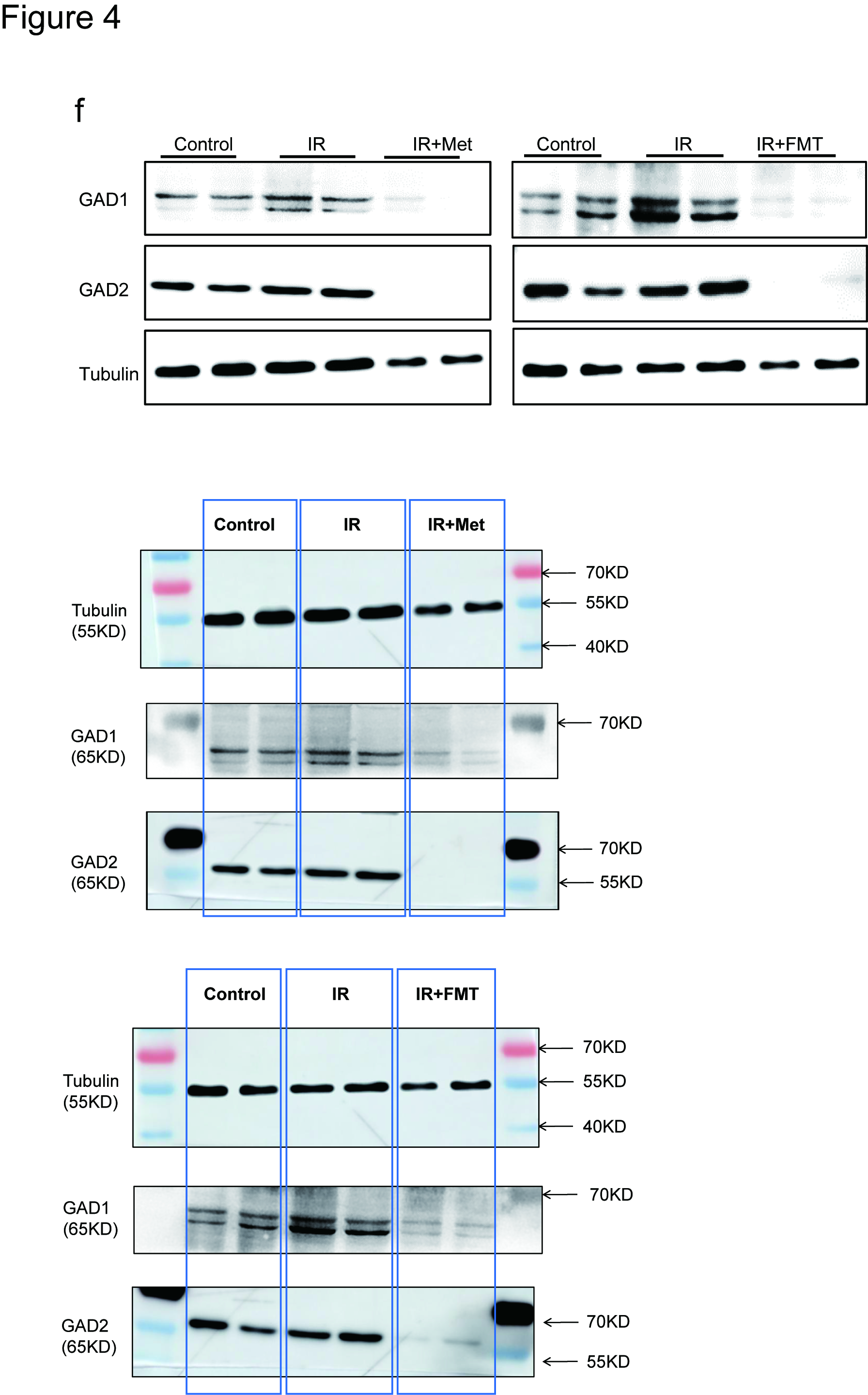

Supplement: Figure 4—source data 1. [file elife-89045-fig4-data1.zip › Figure 4-source data 1.tif]

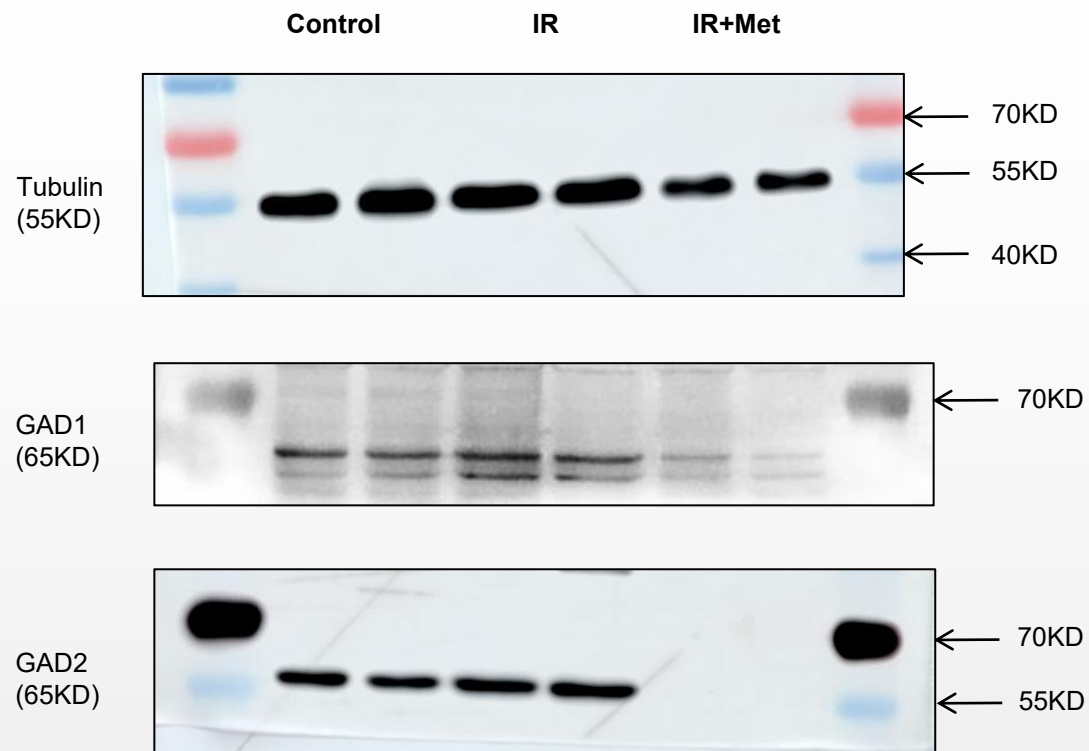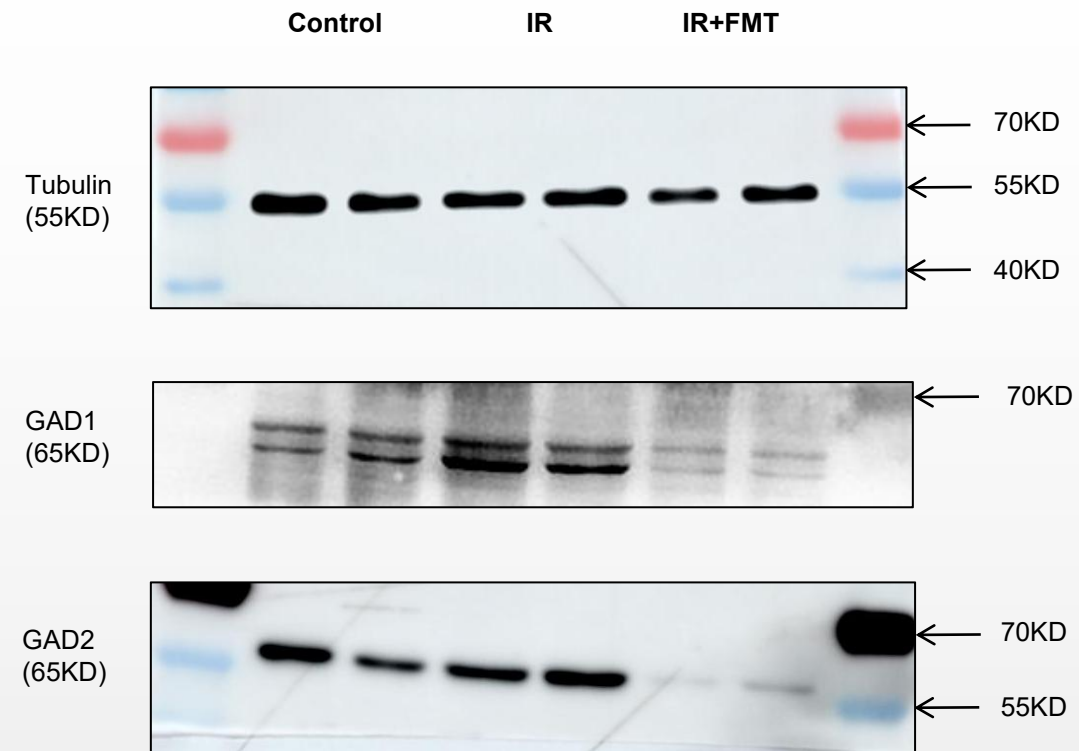

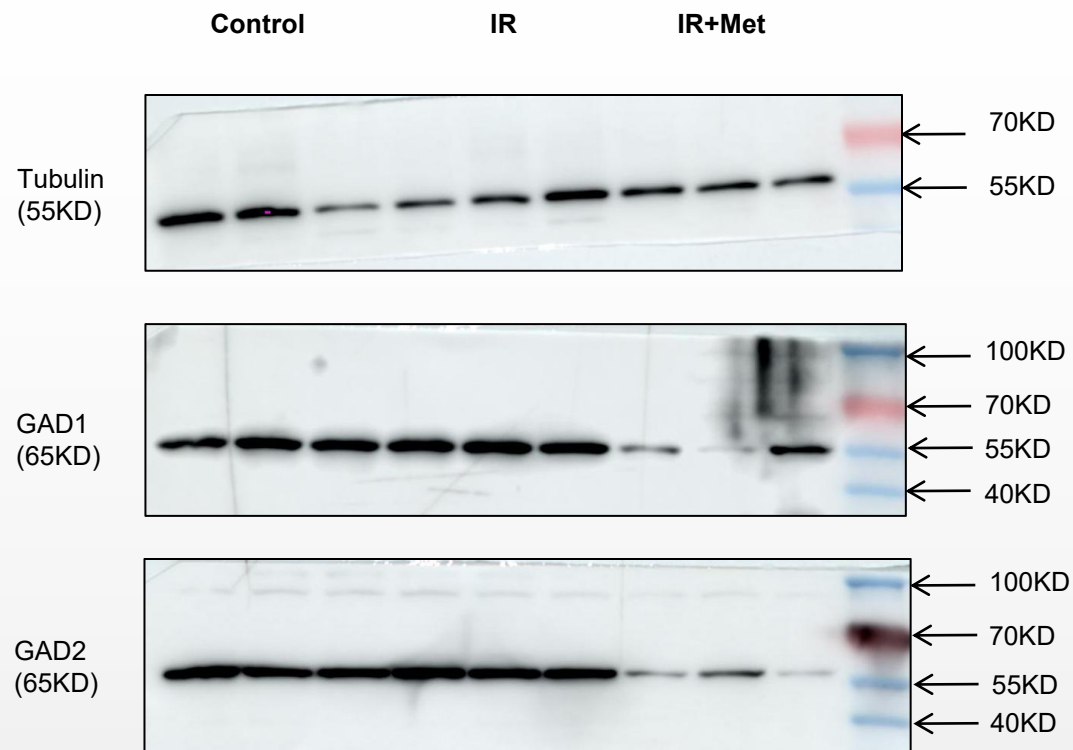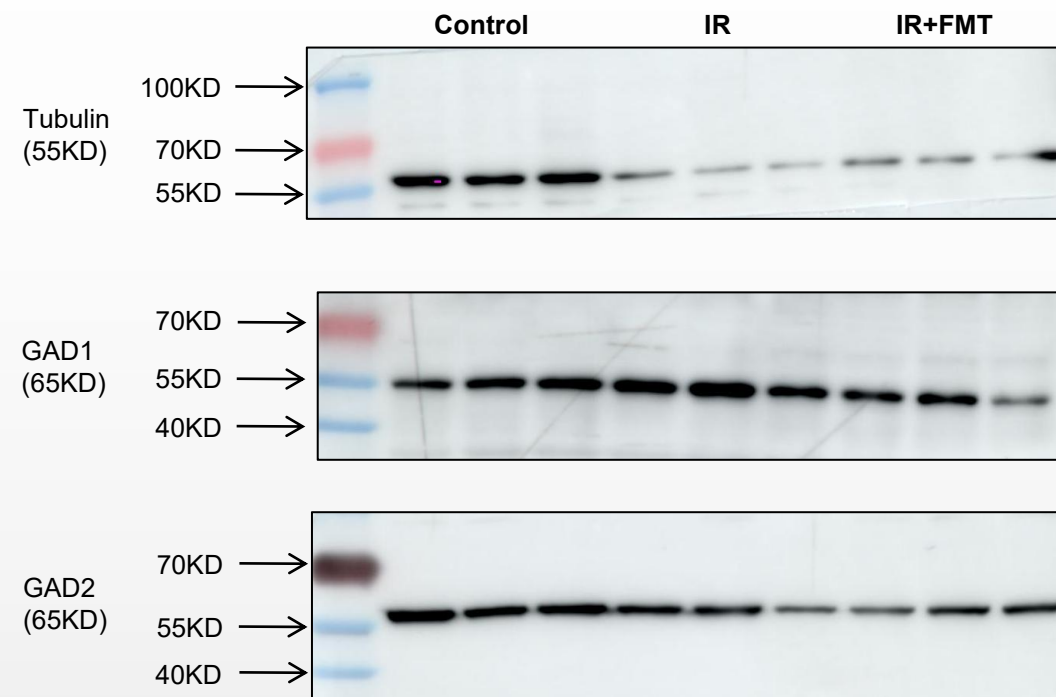

Supplement: Figure 4—source data 2. [file elife-89045-fig4-data2.zip › Figure 4-source data 2.pdf]

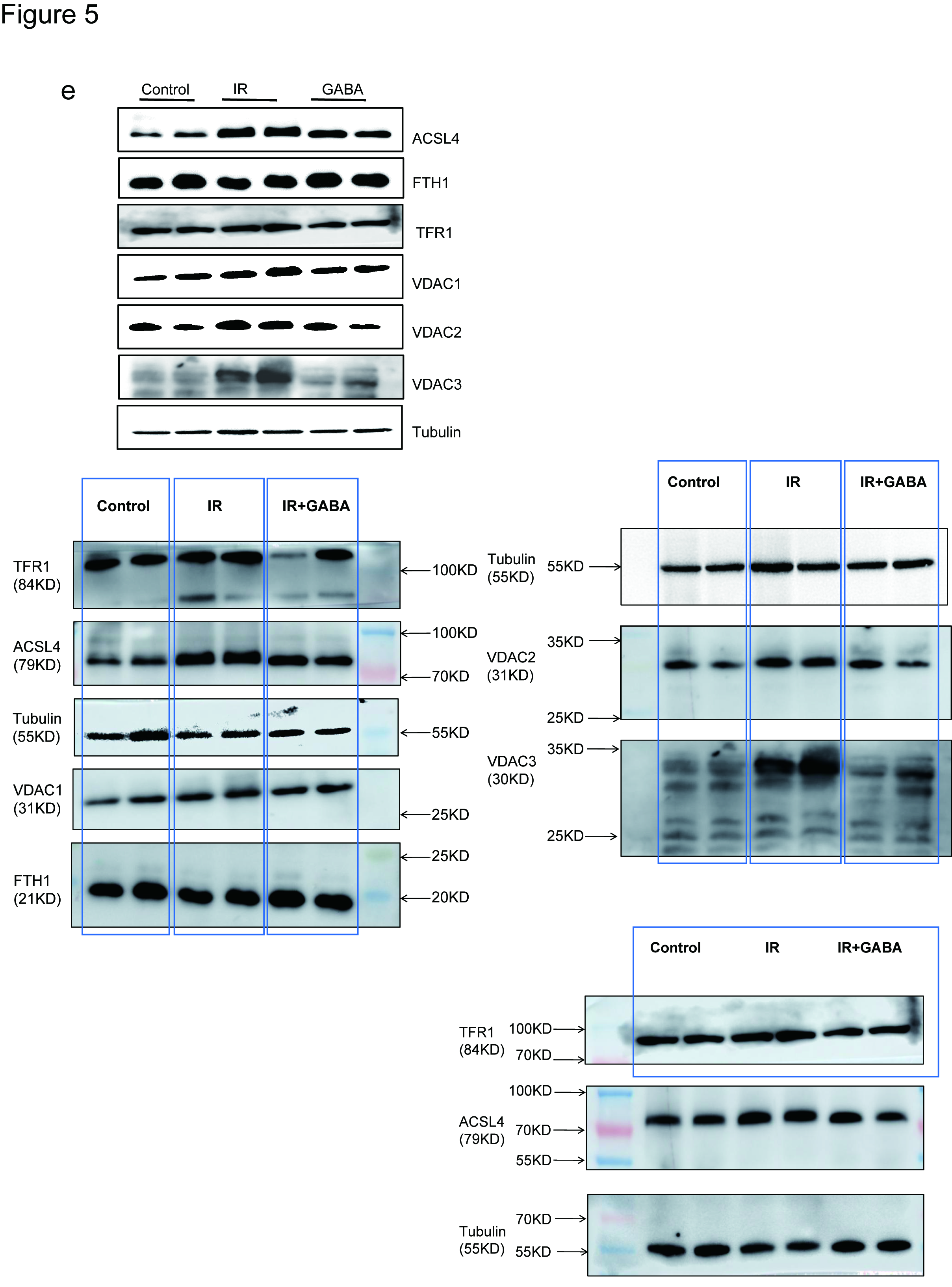

Supplement: Figure 5—source data 1. [file elife-89045-fig5-data1.zip › Figure 5-source data 1.tif]

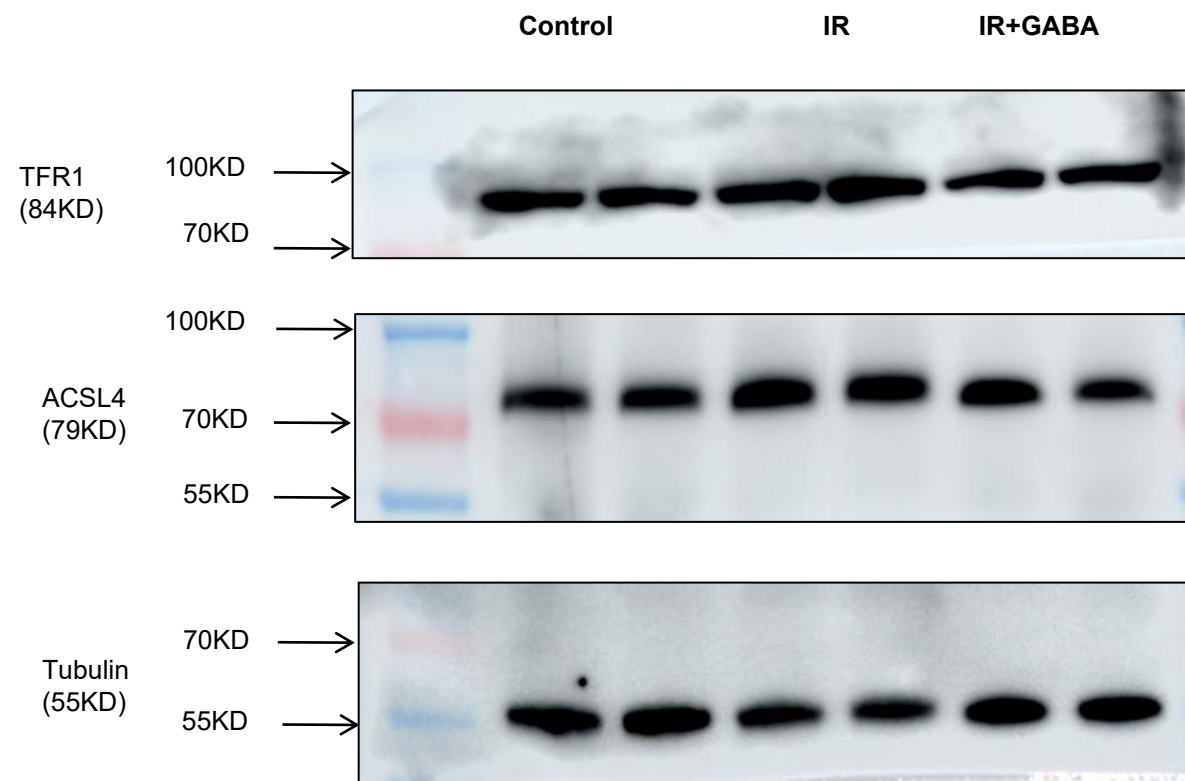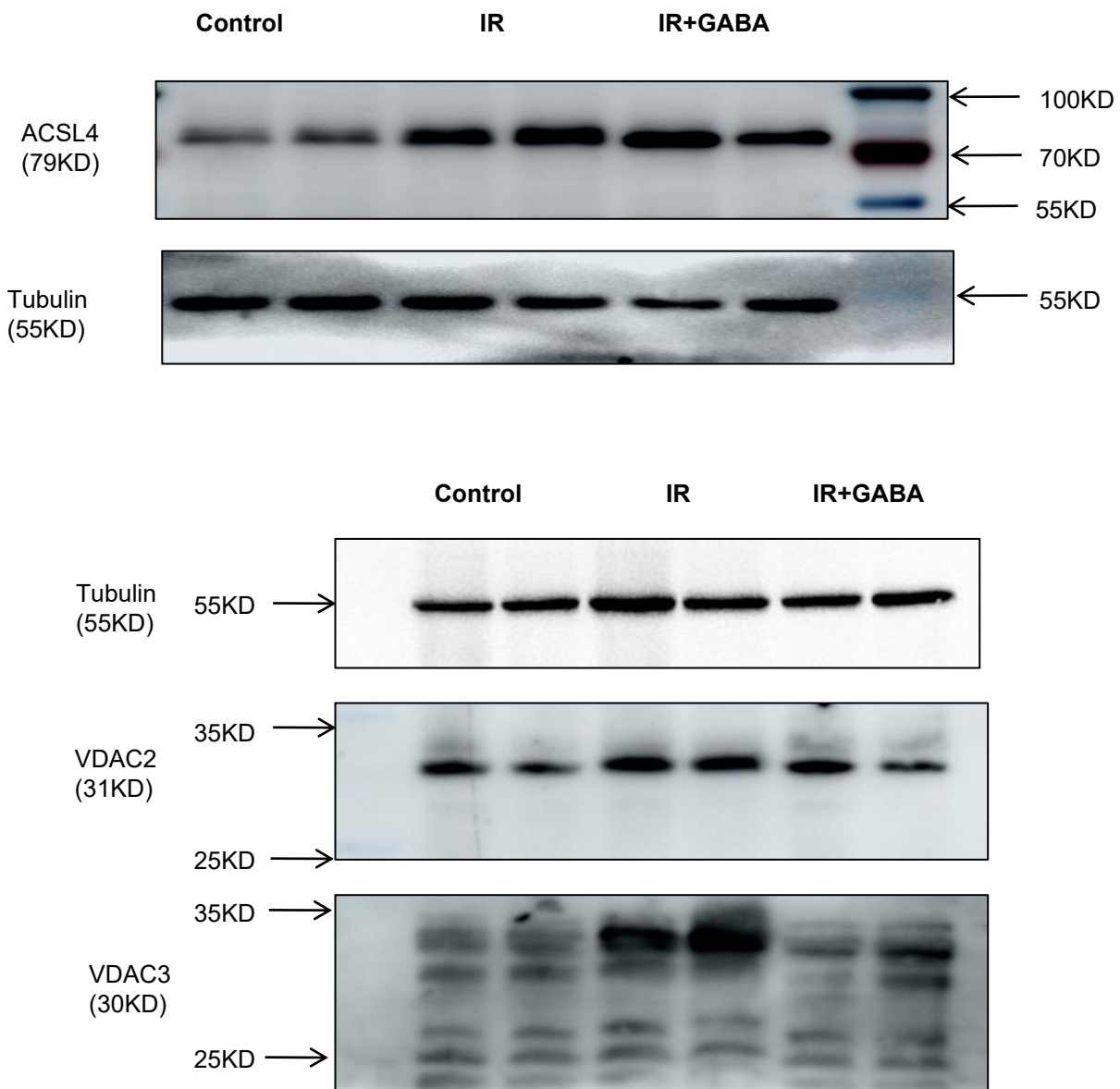

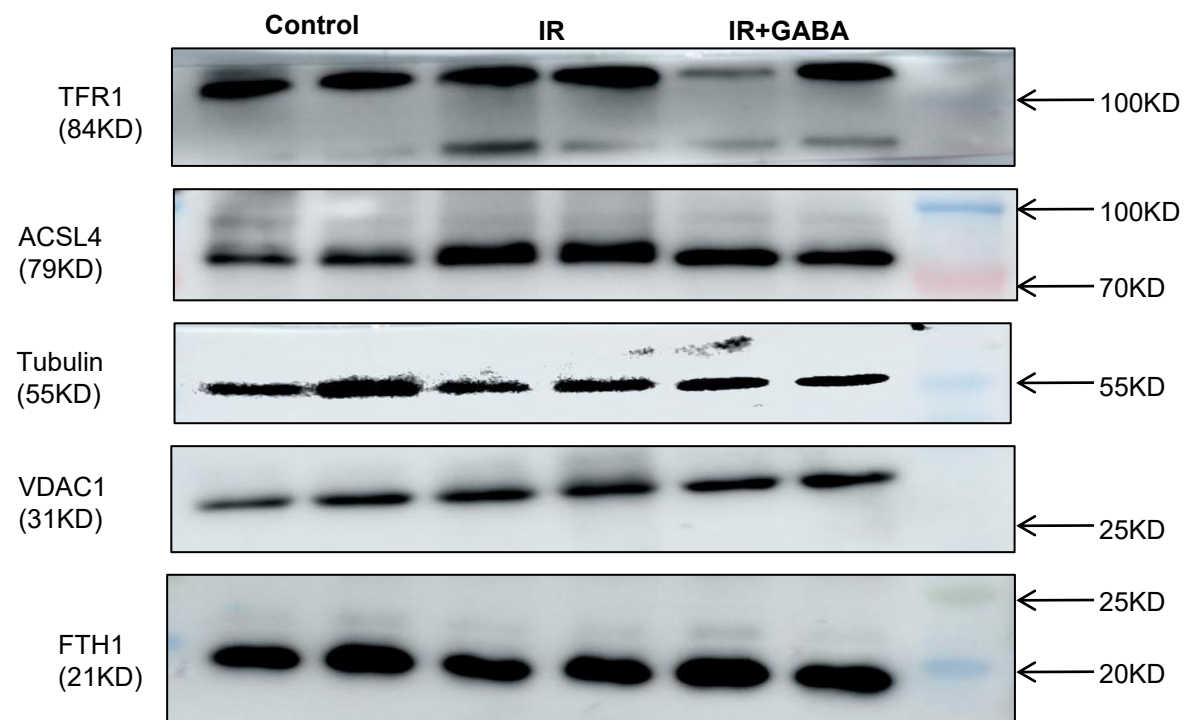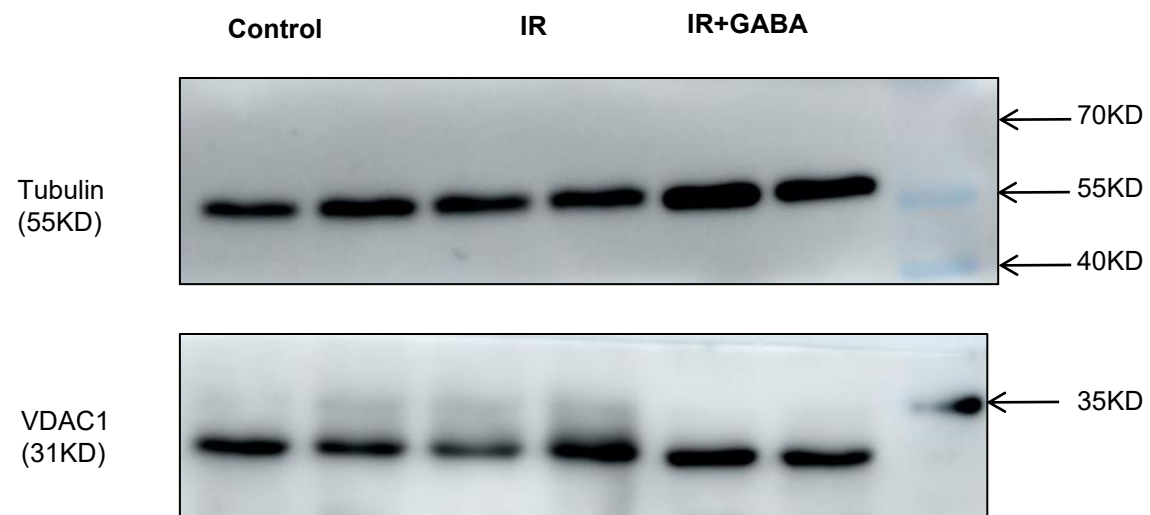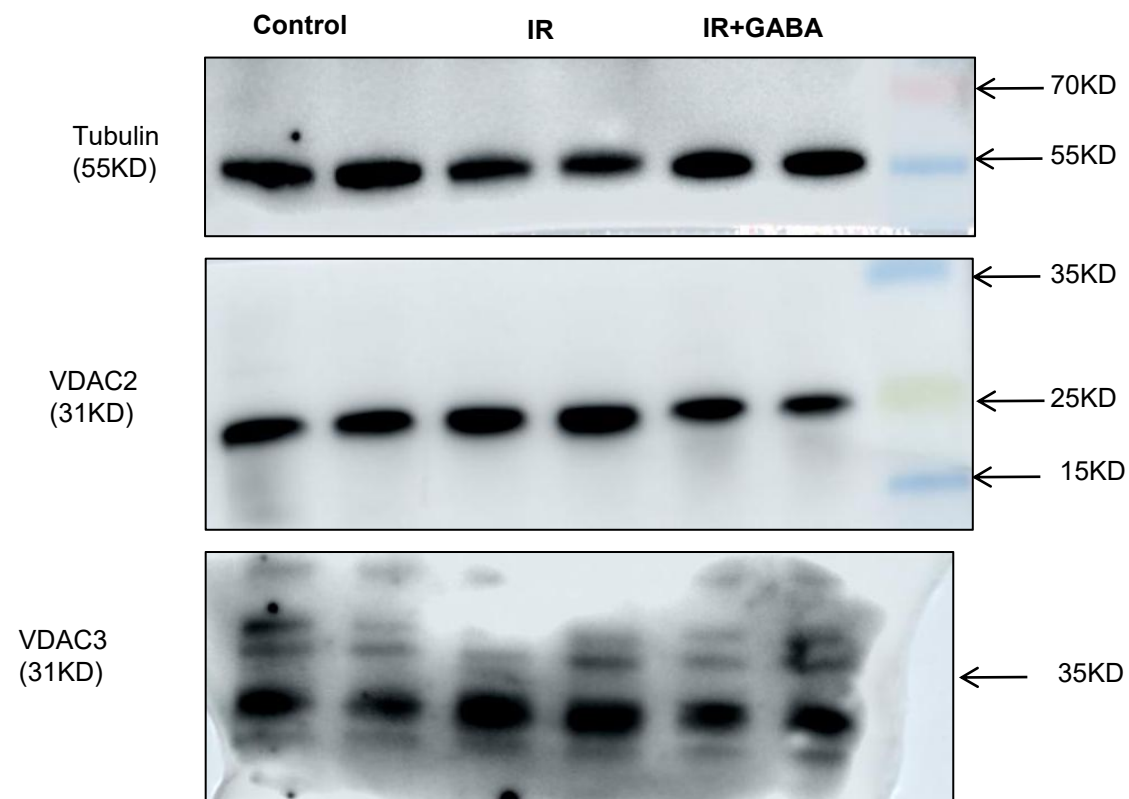

Supplement: Figure 5—source data 2. [file elife-89045-fig5-data2.zip › Figure 5-source data 2.pdf]

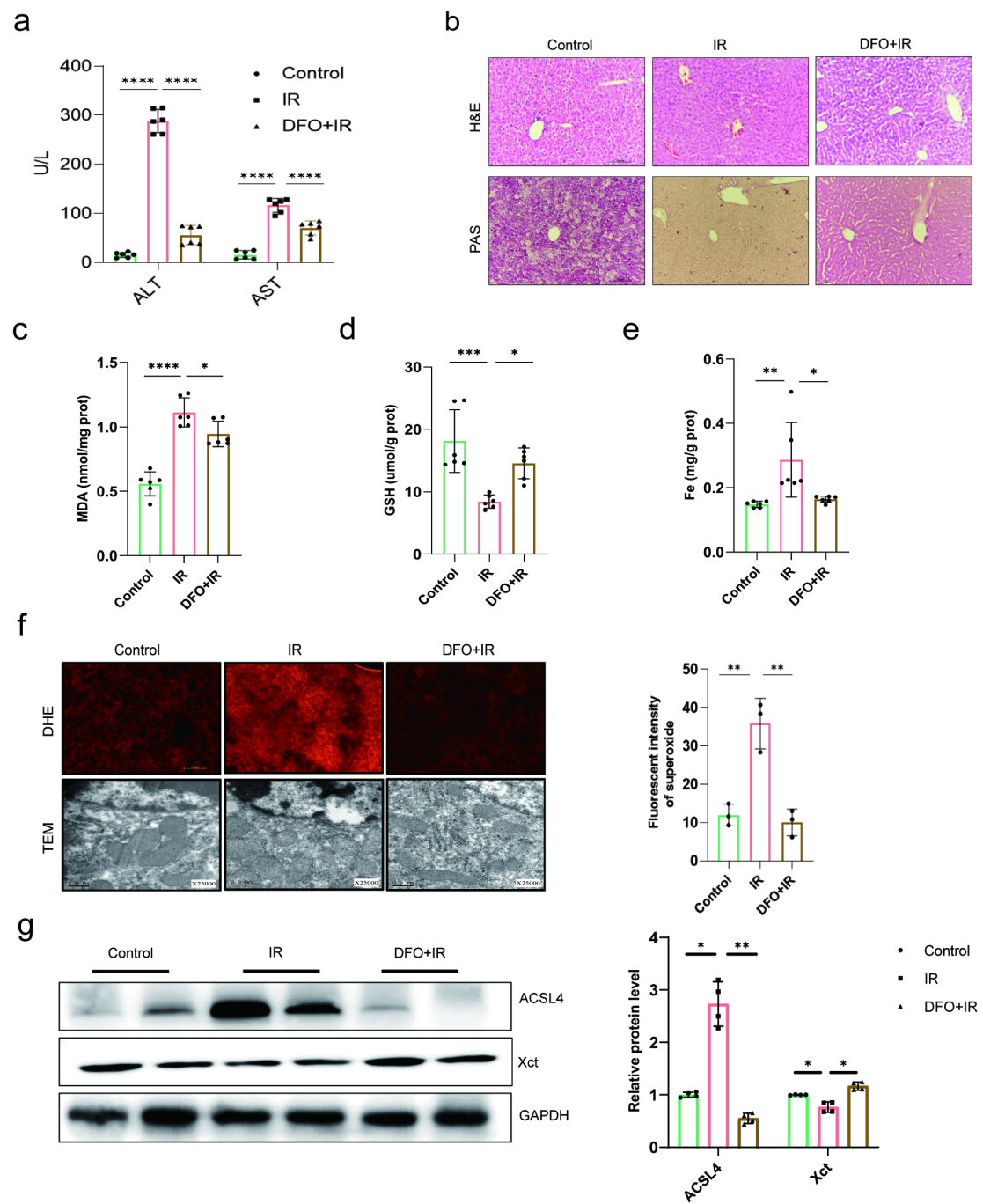

Supplement: Supplementary file 3. [file elife-89045-supp3.pdf]

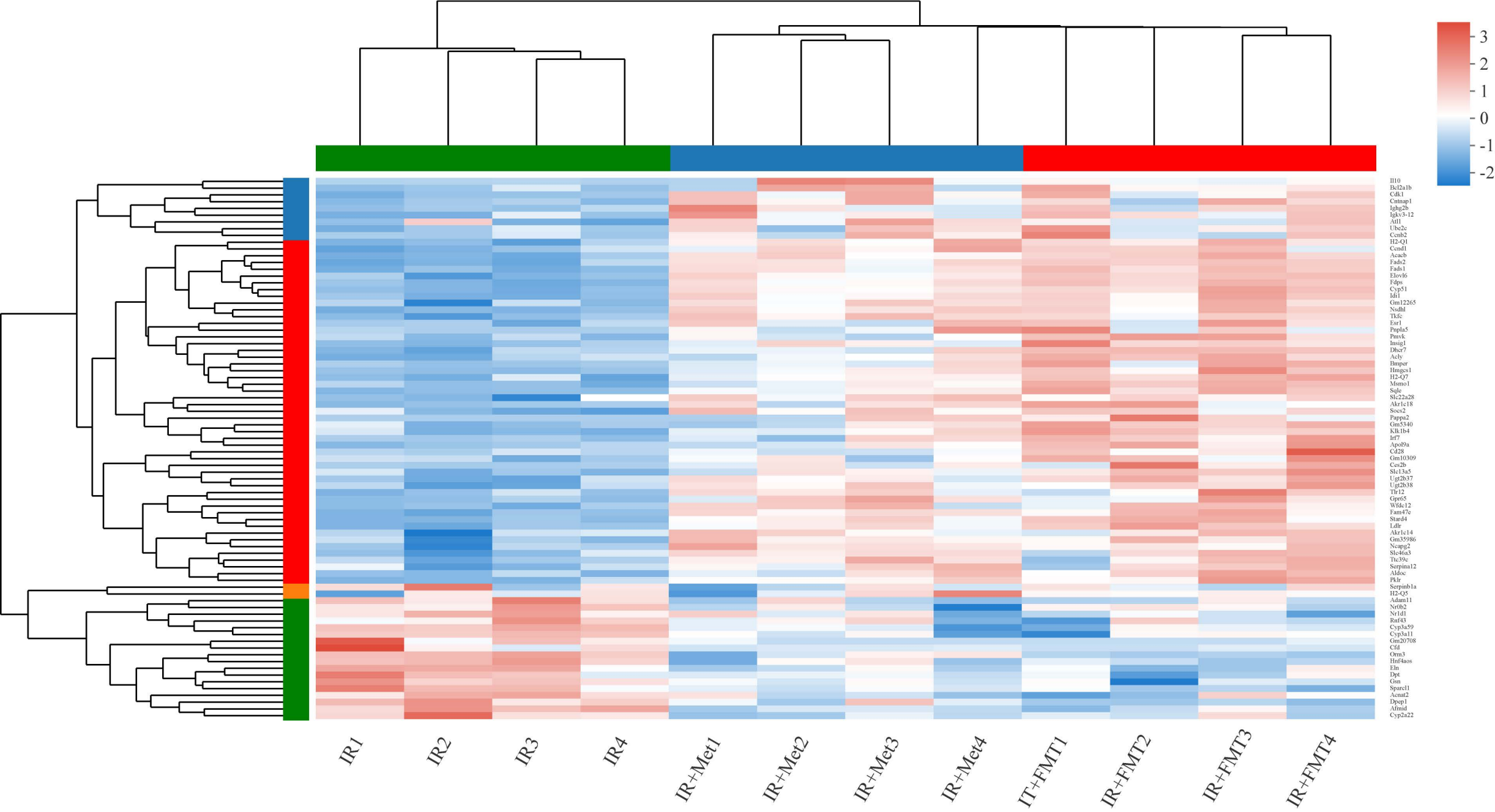

Supplement: Supplementary file 4. [file elife-89045-supp4.pdf]

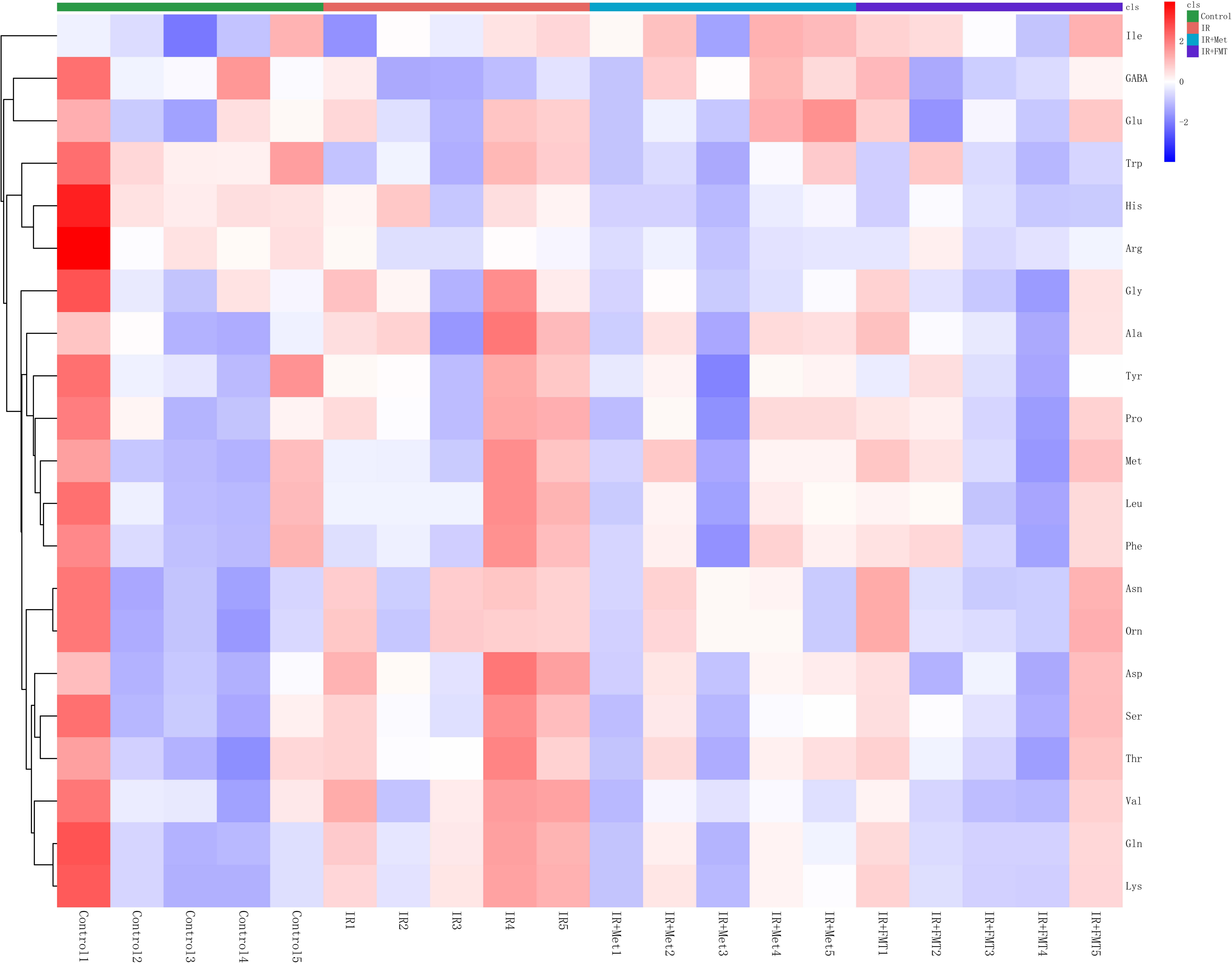

Supplement: Supplementary file 5. [file elife-89045-supp5.pdf]

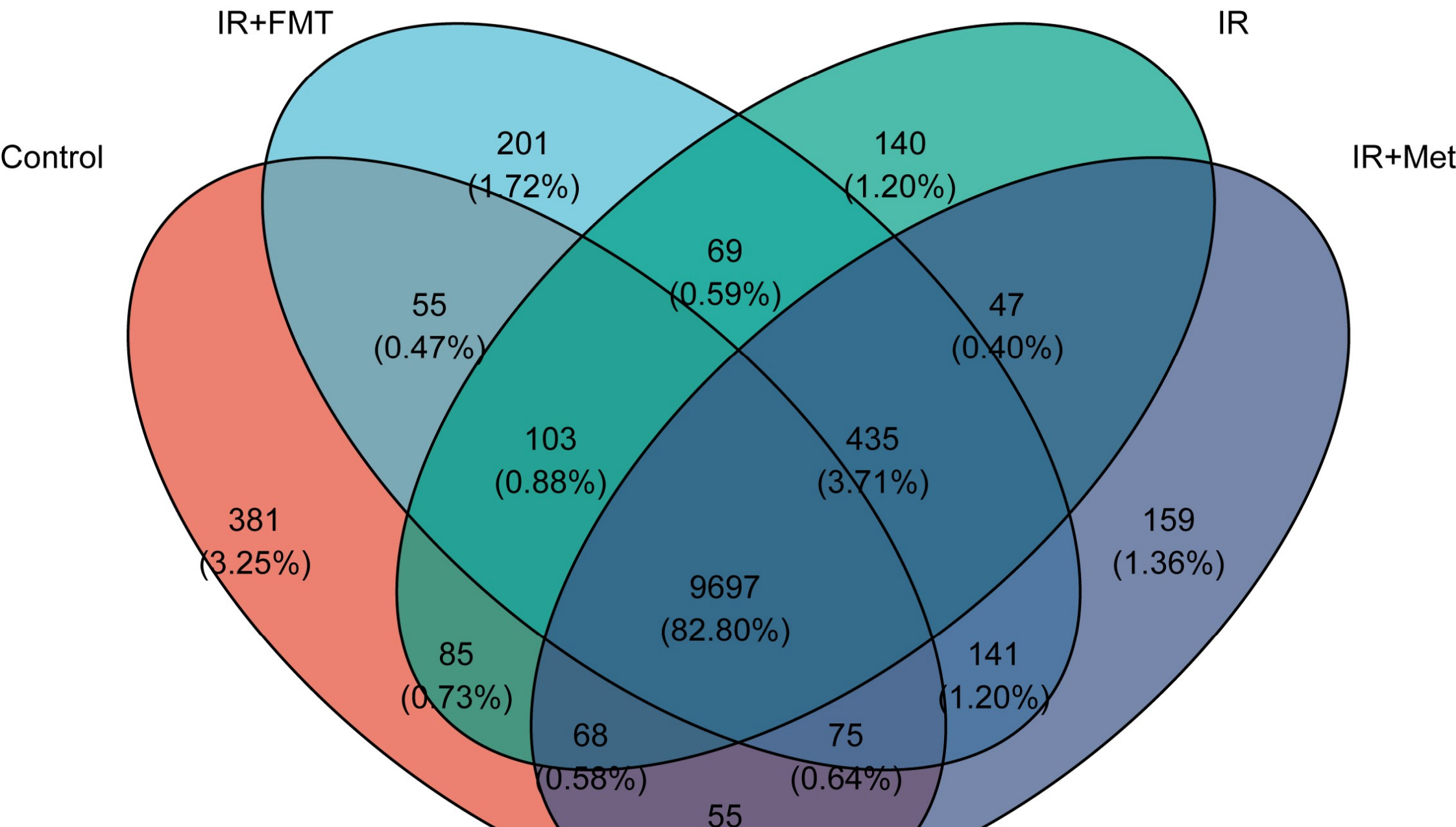

Supplement: Supplementary file 7. [file elife-89045-supp7.pdf]
