# Supplementary material for: Gut microbiota-derived gamma-aminobutyric acid from metformin treatment reduces hepatic ischemia/reperfusion injury through inhibiting ferroptosis
Source: eLife. 2024 Mar 15;12:RP89045. doi: 10.7554/eLife.89045 (PMC10942780; doi:10.7554/eLife.89045)
Supplement: Supplementary file 6. [file elife-89045-supp6.pdf]

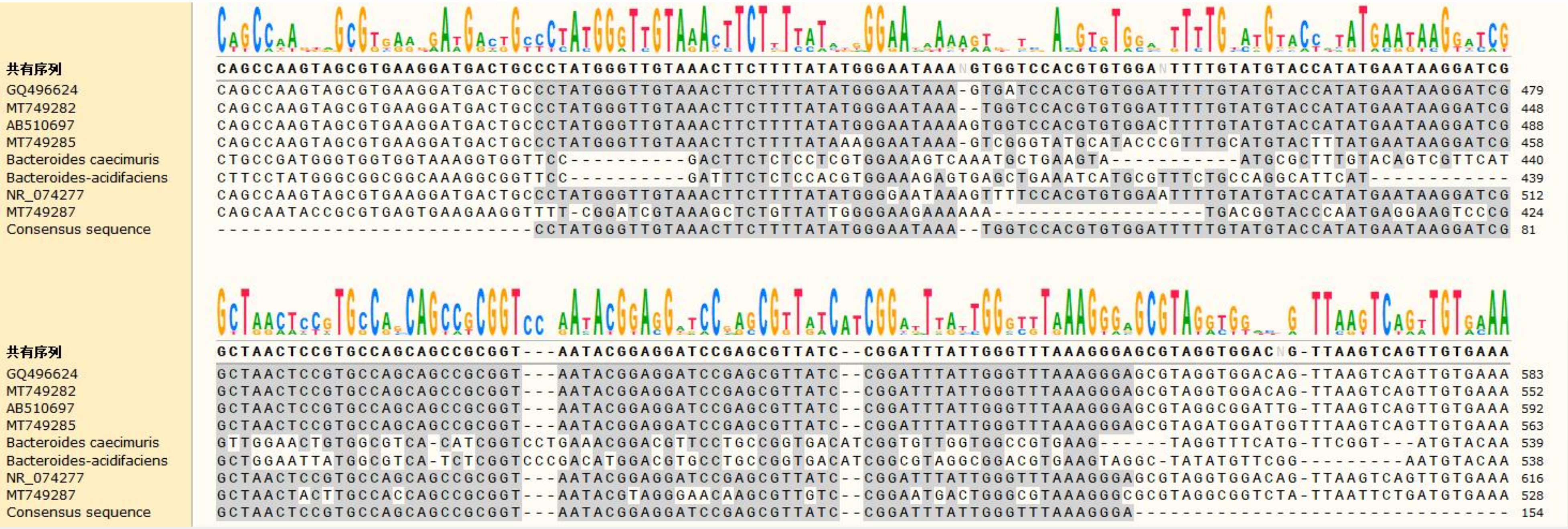
共有序列 CAGCCAAGTAGCGTGAAGGATGACTGCCCTATGGGTTGTAAACTTCTTTTATATGGGAATAAANGTGGTCCACGTGTGGANTTTTGTATGTACCATATGAATAAGGATCG
GQ496624 CAGCCAAGTAGCGTGAAGGATGACTGCCCTATGGGTTGTAAACTTCTTTTATATGGGAATAAA-GTGATCCACGTGTGGATTTTTGTATGTACCATATGAATAAGGATCG 479
MT749282 CAGCCAAGTAGCGTGAAGGATGACTGCCCTATGGGTTGTAAACTTCTTTTATATGGGAATAAA--TGGTCCACGTGTGGATTTTTGTATGTACCATATGAATAAGGATCG 448
AB510697 CAGCCAAGTAGCGTGAAGGATGACTGCCCTATGGGTTGTAAACTTCTTTTATATGGGAATAAAAGTGGTCCACGTGTGGACTTTTGTATGTACCATATGAATAAGGATCG 488
MT749285 CAGCCAAGTAGCGTGAAGGATGACTGCCCTATGGGTTGTAAACTTCTTTTATAAAGGAATAAA-GTCGGGTATGCATACCCGTTTGCATGTACTTTATGAATAAGGATCG 458
Bacteroides caecimuris CTGCCGATGGGTGGTGGTAAAGGTGGTTCC----------GACTTCTCTCCTCGTGGAAAGTCAAATGCTGAAGTA-----------ATGCGCTTTGTACAGTCGTTCAT 440
Bacteroides-acidifaciens CTTCCTATGGGCGGCGGCAAAGGCGGTTCC----------GATTTCTCTCCACGTGGAAAGAGTGAGCTGAAATCATGCGTTTCTGCCAGGCATTCAT------------ 439
NR_074277 CAGCCAAGTAGCGTGAAGGATGACTGCCCTATGGGTTGTAAACTTCTTTTATATGGGGAATAAAGTTTTCCACGTGTGGAATTTTGTATGTACCATATGAATAAGGATCG 512
MT749287 CAGCAATACCGCGTGAGTGAAGAAGGTTTT-CGGATCGTAAAGCTCTGTTATTGGGGAAGAAAAAA------------------TGACGGTACCCAATGAGGAAGTCCCG 424
Consensus sequence ---------------------------CCTATGGGTTGTAAACTTCTTTTATATGGGAATAAA--TGGTCCACGTGTGGATTTTTGTATGTACCATATGAATAAGGATCG 81
共有序列 GCTAACTCCGTGCCAGCAGCCGCGGT---AATACGGAGGATCCGAGCGTTATC--CGGATTTATTGGGTTTAAAGGGAGCGTAGGTGGACNG-TTAAGTCAGTTGTGAAA
GQ496624 GCTAACTCCGTGCCAGCAGCCGCGGT---AATACGGAGGATCCGAGCGTTATC--CGGATTTATTGGGTTTAAAGGGAGCGTAGGTGGACAG-TTAAGTCAGTTGTGAAA 583
MT749282 GCTAACTCCGTGCCAGCAGCCGCGGT---AATACGGAGGATCCGAGCGTTATC--CGGATTTATTGGGTTTAAAGGGAGCGTAGGTGGACAG-TTAAGTCAGTTGTGAAA 552
AB510697 GCTAACTCCGTGCCAGCAGCCGCGGT---AATACGGAGGATCCGAGCGTTATC--CGGATTTATTGGGTTTAAAGGGAGCGTAGGCGGATTG-TTAAGTCAGTTGTGAAA 592
MT749285 GCTAACTCCGTGCCAGCAGCCGCGGT---AATACGGAGGATCCGAGCGTTATC--CGGATTTATTGGGTTTAAAGGGAGCGTAGATGGATGGTTTAAGTCAGTTGTGAAA 563
Bacteroides caecimuris GTTGGAACTGTGGCGTCA-CATCGGTCCTGAAACGGACGTTCCTGCCGGTGACATCGGTGTTGGTGGCCGTGAAG------TAGGTTTCATG-TTCGGT---ATGTACAA 539
Bacteroides-acidifaciens GCTGGAATTATGGCGTCA-TCTCGGTCCCGACATGGACGTGCCTGCCGGTGACATCGGCGTAGGCGGACGTGAAGTAGGC-TATATGTTCGG---------AATGTACAA 538
NR_074277 GCTAACTCCGTGCCAGCAGCCGCGGT---AATACGGAGGATCCGAGCGTTATC--CGGATTTATTGGGTTTAAAGGGAGCGTAGGTGGACAG-TTAAGTCAGTTGTGAAA 616
MT749287 GCTAACTACTTGCCACCAGCCGCGGT---AATACGTAGGGAACAAGCGTTGTC--CGGAATGACTGGGCGTAAAGGGCGCGTAGGCGGTCTA-TTAATTCTGATGTGAAA 528
Consensus sequence GCTAACTCCGTGCCAGCAGCCGCGGT---AATACGGAGGATCCGAGCGTTATC--CGGATTTATTGGGTTTAAAGGGA-------------------------------- 154

```
Bacteroides clarus-2        ATATTCTCGTTCACCGAGGCCAAAGTGCTGG--GCAACTGGACGGGATTCTCCACCAAGCTGTACAGCTCCCTCTTCACG  156
Bacteroides caccae          AGCAAAGCCAGCACTACCACAAAAATAATAGCGGAAAGCGGGCGAAGCTC----------CGGCGTTAAACCACCTTTACG  117
Bacteroides clarus          ATATTCTCGTTCACCGAGGCCAAAGTGCTGG--GCAACTGGACGGGATTCTCCACCAAGCTGTACAGCTCCCTCTTCACG  156
Bacteroides xylanisolvens   ATCTATTCTTTCACCGAAGCGAAAGTGCTGG--GCAACTGGACTGGTTTCTCCACAAAACTTTATACTTCGCTTTTCACT  156
Bacteroides ovatus          AATAACGCCAGTACCACGACAAAAATGATAGCCGAAAGCGGGCGAAGTTC----------CGGGGTCAAACCTCCCTTGCG  117
Bacteroides finegoldii      AGCAATGCCAGCACTACAACAAAAATAATGGCAGAAAGCGGGCGAAGTTC----------CGGTGTCAGCCCTCCTTTACG  117
Bacteroides fragilis        ATTTACTCTTTTACCGAGGCAAAAGTGCTGG--GCAACTGGACCGGATTCTCCACAAAACTGTATTCGTCACTGTTCACT  156
Bacteroides caecimuris      AACAACGCCAGCACTACGACAAAAATAATCGCAGAAAGCGGACGAAGCTC----------CGGTGTCAAACCACCTTTGCG  117
Consensus sequence          ------------------------------------------------------------CGGCGTTAAACCACCTTTACG  21
```

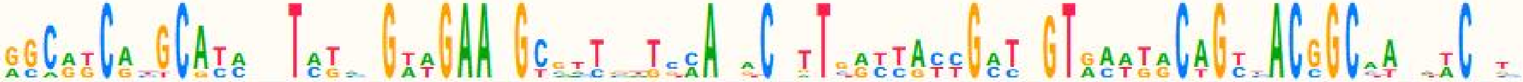

```
共有序列                     GGCATCA-GCATAAATATANGTAGAAAGCGTT--TCCANACCTTGATTACCGATAGTGAATACAGTTACGGCAAAATCAT
Bacteroides clarus-2        GGCGGCATGCACCACTCGCTGATGAACGCCATCTGGAACACATTCGCCATTGCCA-TGCTGGCAGCCACCGCCAGTACGG  235
Bacteroides caccae          GGCATCA-GCATATATATAAGTAGAAAGTGTT--TCCAAGCCTTGGTTACCGATAGTAAATACAGTTACGGCAAAATCAT  194
Bacteroides clarus          GGCGGCATGCACCACTCGCTGATGAACGCCATCTGGAACACATTCGCCATTGCCA-TGCTGGCAGCCACCGCCAGTACGG  235
Bacteroides xylanisolvens   ACAGGCACGCATCATTCTTTGATGAATGCATTGATTAACACGATTACTATTGCTC-TGCTGGCTGCAACGGCATCTACCT  235
Bacteroides ovatus          GGCATCG-GCGTAGATATAAGTAGAAAGCGTT--TCCAAACCTTGATTACCGATAGTGAATACAGTTACGGCAAAATCAT  194
Bacteroides finegoldii      GGCATCG-GCATAAATATAGGTAGAAAGCGTC--TCCAATCCTTGATTGCCGATGGTAAATACAGTCACGGCAAAATCAT  194
Bacteroides fragilis        ACCGGCACTCACCATTCGCTGATGAATGCGCTGATCAATACTGTGACCATTGCTT-TGATTGCAGCTACGGCTTCCACCC  235
Bacteroides caecimuris      AGCATCG-GCATAGATATAAGTAGAAAGCGTC--TCCAGACCTTGATTGCCGATGGTAAATACTGTTACGGCAAAATCAT  194
Consensus sequence          GGCATCA-GCATATATATAAGTAGAAAGTGTT--TCCAAGCCTTGGTTACCGATAGTAAATACAGTTACGGCAAAATCAT  98
```

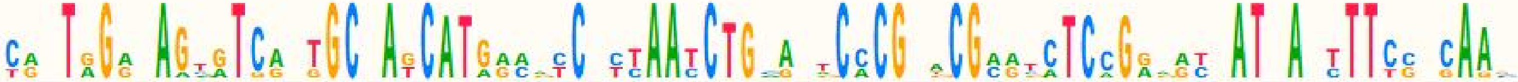

```
共有序列                     CAATGGANAGTGTCANTGCNAGCATGAAACCNCTAATC---ATTCCCGGACGAATCTCNGGAATAATNACTTTCCACAAG
Bacteroides clarus-2        CGTTGGGCAGCATCGCGGCCATC--GGCATCTTCAACCTGCGCACACGCACGCGTC----AGGTGATGAACTTCGCCAAC  309
Bacteroides caccae          CAATAGAGAGTGTCAATGCAAGCATGAAGCCACTAATC---ATTCCCGGACGAACCTCCGGAATAATTACTTTCCACAAG  271
Bacteroides clarus          CGTTGGGCAGCATCGCGGCCATC--GGCATCTTCAACCTGCGCACACGCACGCGTC----AGGTAATGAACTTCGCCAAC  309
Bacteroides xylanisolvens   TGCTGGGTAGTGTGGCTGCTATC--GGCATCTTTAATCTGAAATCCCGTTCGCGTA----AAGCGATTAGTTTTGTGAAT  309
Bacteroides ovatus          CAATGGATAGTGTCAGTGCAAGCATAAAGCCGCTAATC---ATTCCCGGACGAATCTCCGGAATTATGACTTTCCACAAG  271
Bacteroides finegoldii      CAATAGACAGAGTCAATGCCAGCATGAATCCACTAATC---ATTCCCGGACGAATCTCAGGAACAATCACTTTCCACAGG  271
Bacteroides fragilis        TGCTGGGGAGTATCACTGCTATC--GGCATCTTCAACCTGAAGGCACGTTCGCGGA----AGGCCATCAGCTTTGTGAAC  309
Bacteroides caecimuris      CAATAGATAATGTCAATGCAAGCATAAAACCACTAATC---ATTCCCGGGCGAATCTCCGGCACAATGACTTTCCACAAG  271
```
